# Oral iron supplementation and anaemia in children according to schedule, duration, dose and cosupplementation: a systematic review and meta-analysis of 129 randomised trials

**DOI:** 10.1136/bmjgh-2022-010745

**Published:** 2023-02-27

**Authors:** Christopher T Andersen, Daniel M Marsden, Christopher P Duggan, Enju Liu, Dariush Mozaffarian, Wafaie W Fawzi

**Affiliations:** 1 Department of Epidemiology, Harvard University T H Chan School of Public Health, Boston, Massachusetts, USA; 2 The George Washington University School of Medicine and Health Sciences, Washington, DC, USA; 3 Division of Gastroenterology, Hepatology, and Nutrition, Boston Children's Hospital, Boston, Massachusetts, USA; 4 Department of Global Health and Population, Harvard University T H Chan School of Public Health, Boston, Massachusetts, USA; 5 Department of Nutrition, Harvard T H Chan School of Public Health, Boston, Massachusetts, USA; 6 Institutional Centers of Clinical and Translational Research, Boston Children's Hospital, Boston, Massachusetts, USA; 7 Tufts University Friedman School of Nutrition Science and Policy, Boston, Massachusetts, USA; 8 Division of Cardiology, Tufts University School of Medicine, Boston, Massachusetts, USA

**Keywords:** Systematic review, Anaemia, Treatment, Nutrition, Child health

## Abstract

**Introduction:**

WHO guidelines on iron supplementation among children call for further research to identify the optimal schedule, duration, dose and cosupplementation regimen.

**Methods:**

A systematic review and meta-analysis of randomised controlled trials was undertaken. Randomised controlled trials providing ≥30 days of oral iron supplementation versus placebo or control to children and adolescents aged <20 years were eligible. Random-effects meta-analysis was used to summarise the potential benefits and harms of iron supplementation. Meta-regression was used to estimate iron effect heterogeneity.

**Results:**

129 trials with 201 intervention arms randomised 34 564 children. Frequent (3–7/week) and intermittent (1–2/week) iron regimens were similarly effective at decreasing anaemia, iron deficiency and iron deficiency anaemia (p heterogeneity >0.05), although serum ferritin levels and (after adjustment for baseline anaemia) haemoglobin levels increased more with frequent supplementation. Shorter (1–3 months) versus longer (7+ months) durations of supplementation generally showed similar benefits after controlling for baseline anaemia status, except for ferritin which increased more with longer duration of supplementation (p=0.04). Moderate-dose and high-dose supplements were more effective than low-dose supplements at improving haemoglobin (p=0.004), ferritin (p=0.008) and iron deficiency anaemia (p=0.02), but had similar effects to low-dose supplements for overall anaemia. Iron supplementation provided similar benefits when administered alone or in combination with zinc or vitamin A, except for an attenuated effect on overall anaemia when iron was cosupplemented with zinc (p=0.048).

**Conclusions:**

Weekly and shorter duration iron supplementation at moderate or high doses might be optimal approaches for children and adolescents at risk of deficiency.

**Trial registration number:**

CRD42016039948.

**WHAT IS ALREADY KNOWN ON THIS TOPIC**

- Prior meta-analyses have established that paediatric oral iron supplementation is safe and effective for reducing anaemia.
- Consequently, WHO recommends 3 months annually of daily oral iron supplementation for children aged 6 months to 12 years living in regions with a high burden of anaemia.
- However, the 2016 WHO guidelines call for further research to identify the optimal schedule, duration, dose and cosupplementation regimen.

**WHAT THIS STUDY ADDS**

- Our study adds to previous meta-analyses of paediatric iron supplementation by using meta-regression to compare the effect sizes of randomised controlled trials with different schedules, durations, doses and cosupplementation schemes.
- As a result, we are able to marshal a much larger body of literature than prior studies which have been restricted to trials specifically designed to answer questions of effect heterogeneity.

**HOW THIS STUDY MIGHT AFFECT RESEARCH, PRACTICE OR POLICY**

- Our results generally support the WHO recommendations regarding the frequency, duration and dose of iron supplementation.
- However, weekly iron supplementation might be considered as an alternative to the recommended daily regimen in some contexts, given evidence of similar efficacy.
- Furthermore, vitamin A and zinc can be cosupplemented without reducing the benefits of iron.

## Background

Iron deficiency (ID) is the most common micronutrient deficiency globally, with children at particular risk.^1 2^ Among children aged 6–59 months, approximately half of the 273 million cases of anaemia in 2011 were estimated to be due to ID.^3 4^ ID is furthermore a key risk factor for cognitive impairment, impaired immune function and decreased capacity for physical activity.^5–7^

WHO recommends daily oral iron supplementation for all children in regions with a prevalence of anaemia ≥40%. In malaria-endemic settings, iron supplementation is recommended in conjunction with measures to prevent, diagnose and treat malaria.^8^ These recommendations are supported by prior meta-analyses establishing the benefits of iron supplementation in the treatment of anaemia along with safety in the presence of malaria control programmes.^9–12^ However, prior meta-analyses have not investigated the optimal delivery of iron supplementation among children. The 2016 WHO guidelines therefore highlight the need for additional evidence regarding the optimal schedule, duration and dose of iron supplementation, as well as the efficacy of iron supplementation in the presence of cosupplemented micronutrients.^8^

We conducted a systematic review and meta-analysis of randomised controlled trials of oral iron supplementation among children and adolescents aged <20 years and compared the impact of interventions by schedule, duration, dose and cointerventions. Haematologic, anthropometric, infectious and developmental outcomes were included to assess both safety and efficacy.

## Methods

### Search strategy and selection criteria

We adhered to the Cochrane Collaboration’s guidelines for this review.^13^ The protocol was pre-registered with the International Prospective Register of Systematic Reviews (CRD42016039948). Systematic literature searches were performed using PubMed, Scopus, Web of Science and the Cochrane Central Register of Controlled Trials from inception through November 2020. References of eligible articles and previous systematic reviews were additionally reviewed. No language restrictions were placed on the search strategy, although studies were excluded if a translation could not be obtained for those published in a language not spoken by one of the authors. Search terms are presented in online supplemental appendix 1.

10.1136/bmjgh-2022-010745.supp1 Supplementary data

Studies were eligible for inclusion if they met the following criteria:

- Oral iron supplementation was randomly assigned.
- Oral iron supplementation was compared with control or placebo. (Studies comparing multiple doses of iron supplementation and which did not include a group randomised to no iron were excluded due to the lack of a common referent for meta-analysis.)
- Intervention groups differed by iron alone (eg, studies randomising children to iron folate vs placebo were not eligible unless the placebo also included folate, due to potential independent effects of these other components).
- Oral iron supplementation was administered for a minimum period of ≥1 month (≥30 days).
- Participants were aged <20 years.
- Participants did not have any chronic illness (eg, HIV) or belong to a special subpopulation (eg, athletes).

### Data extraction and management

One reviewer screened the titles and abstracts of records identified by the search, and excluded those that indicated clear ineligibility. Two reviewers independently reviewed the full text of all positively screened studies to establish final eligibility, after which data were extracted from eligible studies in duplicate using standardised forms. Online supplemental appendix 2 provides a list of variables extracted from eligible studies. Discrepancies between reviewers were resolved through discussion or through arbitration with a third reviewer. Factorial trials were extracted as two separate experiments (iron vs control, iron+cointervention vs control+cointervention).

### Assessment of risk of bias

Two authors independently assessed risk of bias using the Cochrane Risk of Bias Tool, and discrepancies were resolved by discussion.^13^

### Outcomes

To assess the relative benefits and risks of oral iron supplementation among children and adolescents, we included the following outcomes: haemoglobin (g/L), anaemia (defined by study authors), serum ferritin (μg/L), ID (defined by study authors), iron deficiency anaemia (IDA, defined by study authors); physical growth (height-for-age, weight-for-height, weight-for-age, stunting, wasting and underweight; primarily using the National Center for Health Statistics reference and WHO growth standards and growth reference^14–16^); indicators of infection (diarrhoea, respiratory infection, malaria and intestinal helminths); and child development (standardised means of cognitive, motor and socioemotional domain scores). In order to account for repeated use of the same control group in trials comparing multiple iron treatments to a single control (n=18 trials), the variance of the outcome in the control group was adjusted by dividing the control group sample size by the number of comparisons.

### Statistical analysis

Meta-analysis was conducted for outcomes reported by four or more trials. We used inverse-variance weighted random-effects meta-analysis to account for underlying differences in the trial populations.^17^ Binary outcomes were summarised using risk ratios, prevalence ratios (PR) or rate ratios with 95% CIs. Continuous measures on the same scale were presented using mean differences, and measures reported on different scales using standardised mean differences (SMD). For ferritin, geometric means or medians were included when arithmetic means were not reported. Heterogeneity of effects was measured using the I^2^ statistic. We assessed effect modification using univariate meta-regression for prespecified supplementation variables: schedule (1–2 times/week; 3–7 times/week), duration (1–3 months; 4–6 months; ≥7 months), dose (age-adjusted tertiles; see online supplemental appendix 3) and cointerventions (zinc; vitamin A).^18 19^ In secondary analyses, we explored effect modification by baseline anaemia (all anaemic; all non-anaemic; mixed population of anaemic and non-anaemic; missing baseline anaemia data), child age (0–5 months; 6–23 months; 2–4 years; 5–11 years; 12–19 years), child sex (all female; all male; mixed female and male; missing baseline sex data), WHO region (Africa; Americas; Eastern Mediterranean; Europe; South-East Asia; Western Pacific) and iron formulation (ferrous sulfate; ferrous fumarate; other or unspecified). We conducted multivariate meta-regression to investigate collinearity between potential effect modifiers.^20^ Small study effects were assessed using funnel plots and Egger’s test for all outcomes reported by ≥10 iron intervention groups. Sensitivity analyses correcting for small study effects were conducted using trim and fill.^21^

### Role of the funder

The funder was not involved in the study design, data collection, analysis, interpretation or manuscript writing. The corresponding author had access to all data in the study and assumes full responsibility for the accuracy of the results.

### Patient and public involvement

Our prior clinical and research experiences with iron-deficient paediatric patients led us to develop the idea for this study. No patients were involved in the meta-analysis design or conduct, though we aim to present findings of clinical relevance to patients. We anticipate that the results of this study would be of interest to clinicians and health policy makers globally, which could in turn contribute to improved patient care.

## Results

### Literature search

**Figure 1:**
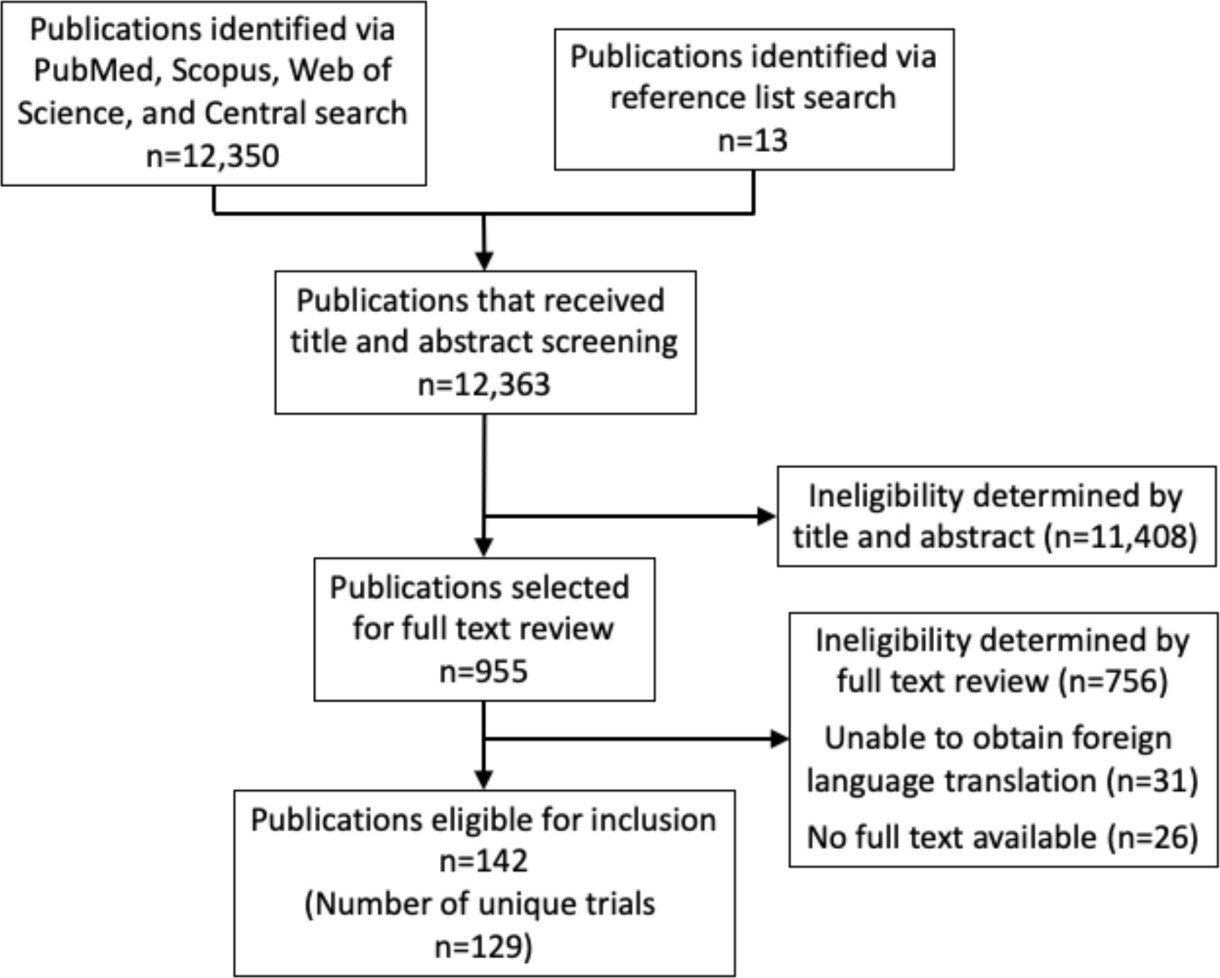
Flow diagram for selection process of eligible studies.

A total of 12 350 unique publications were retrieved from PubMed, Scopus, Web of Science and Cochrane Central search. An additional 13 records identified from the reference lists of eligible articles or prior meta-analyses were also screened. Of these, 955 records were selected for full text review, of which 79 required discussion between independent reviewers to determine eligibility, and 13 required arbitration from a third coauthor. One hundred and forty-two studies met the final eligibility criteria for inclusion (figure 1). Online supplemental appendix 4 provides references to all included publications.

### Trial characteristics

The 142 eligible publications represented 129 unique trials with 201 trial arms randomised to iron supplementation and 177 trial arms randomised to control or placebo, and 34 564 total participants analysed (table 1). Children under 5 years of age were most frequently studied, with children aged <6 months (n=45 trial arms, 22%), 6–23 months (n=44, 22%) and 2 to <5 years (n=37, 18%) representing roughly equal proportions of this population. School-age children of 5–11 years (n=50, 25%) and adolescents aged 12–19 years (n=25, 12%) were less frequently studied than children under 5. About half of studies were in anaemic only (n=36, 18%) or mixed anaemic and non-anaemic (n=70, 35%) children, and about 1 in 6 (n=32, 16%) in non-anaemic children; the remainder (n=63, 32%) did not report baseline anaemia status. Most studies (n=163, 81%) provided iron supplementation 3–7 days/week, although many (n=35, 17%) supplemented only 1–2 days/week. About half of studies (n=113, 56%) provided supplementation for 1–3 months; about one-third (n=62, 31%) for 4–6 months; and the remainder (n=26, 13%) for ≥7 months. About one-fifth (n=30, 23%) of eligible studies were factorial trials, among which the most frequent cointerventions were zinc (n=11) and vitamin A (n=6). The characteristics of individual studies are available in online supplemental appendix 5.

**Table 1:** Characteristics of studies included in meta-analysis of randomised iron supplementation trials for child health

Study characteristics	
Eligible publications, n	142
Unique trials, n	129
Trial arms randomised to iron, n	201
Trial arms randomised to placebo or control, n	177
Individuals randomised to iron, n	18 142
Individuals randomised to placebo or control, n	16 422
WHO region, n (%)*	
Africa	40 (19.9)
Americas	36 (17.9)
Eastern Mediterranean	12 (6.0)
Europe	26 (12.9)
South-East Asia	67 (33.3)
Western Pacific	20 (10.0)
Decade of publication, n (%)†	
1970–1979	3 (2.1)
1980–1989	17 (12.0)
1990–1999	26 (18.3)
2000–2009	69 (48.6)
2010–2017	27 (19.0)
**Population characteristics**	
Age, n (%)*	
0–5 months	45 (22.4)
6–23 months	44 (21.9)
2 to <5 years	37 (18.4)
5 to <12 years	50 (24.9)
≥12 years	25 (12.4)
Per cent female, median (IQR)*	50.4 (47.0, 55.2)
All female, n (%)	28 (13.9)
All male, n (%)	6 (3.0)
Mixed male and female, n (%)	125 (61.2)
Missing baseline sex data, n (%)	42 (20.9)
Baseline per cent anaemic, median (IQR)*	55.2 (9.1, 100.0)
All anaemic, n (%)	36 (17.9)
All non-anaemic, n (%)	32 (15.9)
Mixed anaemic and non-anaemic, n (%)	70 (34.8)
Missing baseline anaemia data, n (%)	63 (31.3)
**Intervention characteristics**	
Frequency, n (%)*	
1–2 days/week	35 (17.4)
3–7 days/week	163 (81.1)
Missing frequency data	3 (1.5)
Duration, n (%)*	
1–3 months	113 (56.2)
4–6 months	62 (30.9)
≥7 months	26 (12.9)
Weekly iron dose (mg) by child age category, median (IQR)*	
0–5 months	52.5 (49.0, 70.0)
6–23 months	70.0 (47.5, 162.8)
24–59 months	105.0 (60.0, 210.0)
5–11 years	265.1 (120.0, 403.1)
12–19 years	205.0 (85.0, 325.0)
Missing dose information, n	28
Formulation, n (%)*	
Ferrous sulfate	122 (60.7)
Ferrous fumarate	20 (10.0)
Other or unspecified	59 (27.7)
Factorial trials, n (%)‡	30 (23.3)
Zinc, n (%)§	11 (36.7)
Vitamin A, n (%)§	6 (20.0)
Other, n (%)§	13 (43.3)

*Denominator is the number of unique groups randomised to iron (n=201).

†Denominator is the number of publications (n=142).

‡Denominator is the number of unique trials (n=129).

§Denominator is the number of factorial trials (n=30).

### Main effects of iron supplementation on haematologic outcomes

In aggregate, oral iron supplementation versus placebo or control demonstrated clear benefits for haematologic indices (table 2). Haemoglobin levels rose by 6.3 g/L (95% CI 5.5, 7.1) along with serum ferritin increases of 18.5 ng/mL (16.1, 20.9). Iron supplementation reduced the prevalence of overall anaemia by 39% (33%, 45%), and even larger impacts were observed for ID (reduction of 70% (63%, 76%)) and IDA (reduction of 80% (69%, 87%)). Heterogeneity was observed between studies for these haematologic outcomes (I^2^ ranging from 80% to 100%), which was further explored using meta-regression (see below).

**Table 2:** Effect of oral iron supplementation versus placebo or control among children and adolescents aged <20 years

	n*	Estimate type	Estimate of effect (95% CI)	P value	I^2^ (%)
Haematology					
Haemoglobin (g/L)	167	WMD	6.3 (5.5 to 7.1)	<0.001	94.1
Serum ferritin (ng/mL)	107	WMD	18.5 (16.1 to 20.9)	<0.001	99.5
Anaemia	69	RR	0.61 (0.55 to 0.67)	<0.001	85.8
Iron deficiency	48	RR	0.30 (0.24 to 0.37)	<0.001	90.1
Iron deficiency anaemia	27	RR	0.20 (0.13 to 0.31)	<0.001	79.7
Anthropometry					
Height-for-age Z score	43	WMD	0.00 (−0.03 to 0.03)	0.99	33.9
Weight-for-height Z score	26	WMD	0.01 (−0.05 to 0.08)	0.71	71.7
Weight-for-age Z score	43	WMD	0.01 (−0.04 to 0.05)	0.78	64.7
Stunting	13	RR	1.07 (0.96 to 1.18)	0.22	0
Wasting	6	RR	1.12 (0.85 to 1.48)	0.42	0
Infections					
Diarrhoea (cumulative incidence)	15	RR	0.97 (0.84 to 1.11)	0.63	0
Diarrhoea (incidence rate)	8	IRR	1.08 (0.98 to 1.19)	0.10	63.4
Respiratory illness (cumulative incidence)	8	RR	1.16 (0.93 to 1.45)	0.20	66.2
Respiratory illness (incidence rate)	9	IRR	0.98 (0.92 to 1.06)	0.66	0
Malaria (prevalence)	12	PR	1.12 (0.99 to 1.25)	0.07	0
Malaria (incidence rate)	7	IRR	0.91 (0.82 to 1.01)	0.08	0
Hookworm (prevalence)	4	PR	0.94 (0.85 to 1.03)	0.19	0
*Ascaris lumbricoides* (prevalence)	4	PR	1.04 (0.88 to 1.25)	0.68	0
*Trichuris trichiura* (prevalence)	4	PR	0.97 (0.90 to 1.06)	0.52	0
Development					
Bayley Mental Index	9	SMD	0.26 (0.00 to 0.51)	0.05	67.8
Bayley Psychomotor Index	9	SMD	0.21 (−0.06 to 0.48)	0.13	70.4

*Number of trial arms randomised to iron.

IRR, incidence rate ratio; PR, prevalence ratio; RR, risk ratio; SMD, standardised mean difference; WMD, weighted mean difference.

### Assessment of heterogeneity for haematologic outcomes

When comparing trials of frequent (3–7 times/week) versus intermittent (1–2 times/week) supplementation, no significant differences in treatment effects were observed for haemoglobin, anaemia, ID and IDA outcomes (table 3). However, trials of frequent supplementation achieved larger increases in serum ferritin than trials of intermittent supplementation (20.9 ng/mL (18.2, 23.7) vs 6.7 ng/mL (3.7, 9.6); p interaction <0.001). After control for baseline anaemia in the study population, frequent supplementation was associated with a larger increase in both ferritin and haemoglobin levels as compared with intermittent supplementation (online supplemental appendix 6).

**Table 3:** Comparison of iron supplementation effects across schedule, duration and dosing schemes

	Haemoglobin (g/L)*	Ferritin (ng/mL)*	Anaemia†	Iron deficiency†	Iron deficiency anaemia†
**Weekly frequency**					
Frequent (3–7 times/week)					
n	132	88	57	44	24
Estimate	6.6	20.9	0.62	0.31	0.20
95% CI	(5.6 to 7.6)	(18.2 to 23.7)	(0.56 to 0.69)	(0.24 to 0.38)	(0.13 to 0.32)
Intermittent (1–2 times/week)					
n	32	18	10	4	3
Estimate	4.8	6.7	0.61	0.24	0.22
95% CI	(3.4 to 6.2)	(3.7 to 9.6)	(0.41 to 0.92)	(0.09 to 0.66)	(0.06 to 0.77)
P value for interaction	0.14	<0.001	0.87	0.73	0.91
**Duration**‡					
1–3 months					
n	96	59	27	18	6
Estimate	7.3	13.9	0.53	0.38	0.23
95% CI	(6.0 to 8.6)	(11.7 to 16.1)	(0.45 to 0.62)	(0.25 to 0.57)	(0.07 to 0.81)
4–6 months					
n	53	41	34	26	19
Estimate	5.7	25.1	0.63	0.21	0.15
95% CI	(4.3 to 7.0)	(20.0 to 30.1)	(0.54 to 0.72)	(0.15 to 0.30)	(0.11 to 0.22)
≥7 months					
n	18	7	8	4	2
Estimate	2.6	11.8	0.84	0.84	0.86
95% CI	(1.4 to 3.8)	(5.9 to 17.7)	(0.69 to 1.03)	(0.67 to 1.06)	(0.63 to 1.18)
P value for interaction	0.01	0.01	0.07	0.05	<0.001
**Dose**					
Low (1st tertile for age)					
n	59	41	27	17	13
Estimate	4.4	13.7	0.68	0.33	0.26
95% CI	(3.2 to 5.6)	(9.5 to 17.9)	(0.57 to 0.81)	(0.22 to 0.50)	(0.17 to 0.41)
Moderate (2nd tertile for age)					
n	32	22	23	17	10
Estimate	8.6	26.0	0.55	0.27	0.08
95% CI	(5.9 to 11.4)	(19.0 to 32.9)	(0.47 to 0.66)	(0.18 to 0.40)	(0.05 to 0.14)
High (3rd tertile for age)					
n	37	25	9	9	2
Estimate	7.2	16.2	0.64	0.24	0.08
95% CI	(5.3 to 9.1)	(13.4 to 19.0)	(0.49 to 0.84)	(0.12 to 0.47)	(0.01 to 0.69)
P value for interaction	0.004	0.008	0.44	0.82	0.02

*Weighted mean difference.

†Pooled risk ratio.

‡Trials of short duration were more likely to have a higher proportion of patients with anaemia. No significant associations were observed between duration and haemoglobin, anaemia, iron deficiency and iron deficiency anaemia outcomes after controlling for baseline anaemia. After control for anaemia, an increase in serum ferritin was associated with trials of longer duration (see online supplemental appendix 5).

Increasing duration of supplementation was generally associated with diminished impacts on haemoglobin, ID and IDA (table 3). However, trials of short duration were more likely to have a higher proportion of patients with anaemia, which could lead to larger effects. After controlling for baseline anaemia, no significant associations were observed between duration of supplementation and haemoglobin, anaemia, ID or IDA outcomes. Trials of longer duration were associated with greater increases in serum ferritin after adjustment for baseline anaemia (online supplemental appendix 6).

When we evaluated age-adjusted doses of supplementation (see online supplemental appendix 3 for the age-specific dose categories), trials in the lowest dose tertile across all ages used lower amounts than recommended by WHO (10–12.5 mg/day for ages 6–23 months, 30 mg/day for ages 24–59 months and 30–60 mg/day for ages 5–12 years).^8^ Compared with lower age-adjusted doses of supplementation and adjusting for baseline anaemia, moderate doses were associated with greater improvements in haemoglobin, ferritin and IDA (table 3; online supplemental appendix 6). However, the lower dose still produced benefits; and no linear dose–response was seen: compared with lower dose, the highest doses were not associated with significantly greater effects.

When comparing the effect of iron stratified by the baseline prevalence of anaemia, trials conducted among entirely anaemic populations demonstrated approximately twofold increases in haemoglobin (global p interaction <0.001) and reductions in endline anaemia (global p interaction=0.004) relative to non-anaemic populations (online supplemental appendix 7). Heterogeneity was observed for some outcomes by child age and sex, although there was no consistent pattern. Effects of iron on haematologic outcomes were similar across WHO regions, except for the impact on IDA (global p interaction=0.007). Comparing types of supplements, ferrous sulfate was associated with the largest increases in haemoglobin (p=0.02) and serum ferritin (p=0.005) and the largest reduction in IDA (p<0.001) compared with other iron formulations.

### Cosupplementation

In factorial trials of iron and zinc supplementation, a borderline interaction was seen for iron effects on the prevalence of anaemia, with stronger reductions in anaemia among children not receiving zinc (PR=0.41 (0.33, 0.50)) versus those receiving zinc (PR=0.64 (0.48, 0.84)) (p interaction=0.048) (table 4). No significant differences by zinc cosupplementation were seen for haemoglobin, ferritin, ID or IDA, although effects generally appeared qualitatively stronger without zinc cosupplementation for each of these outcomes. Zinc alone provided no statistically significant benefits for haematologic outcomes (online supplemental appendix 8). There was no statistically significant difference in the effect of iron supplementation when administered with or without vitamin A. Vitamin A alone improved haemoglobin and anaemia outcomes (online supplemental appendix 8).

**Table 4:** Comparison of iron supplementation effects within factorial trials

	Haemoglobin (g/L)*	Ferritin (ng/mL)*	Anaemia†	Iron deficiency†	Iron deficiency anaemia†
Zinc					
Iron+zinc versus zinc					
n	10	9	6	6	5
Estimate	4.2	21.1	0.64	0.18	0.15
95% CI	(1.5 to 6.9)	(16.0 to 26.1)	(0.48 to 0.84)	(0.12 to 0.28)	(0.09, 0.24)
Iron versus control/placebo					
n	10	9	6	6	5
Estimate	6.6	28.8	0.41	0.15	0.08
95% CI	(3.6 to 9.6)	(22.2 to 35.4)	(0.33 to 0.50)	(0.09 to 0.24)	(0.05, 0.15)
P value for interaction	0.27	0.19	0.048	0.62	0.13
Vitamin A					
Iron+vitamin A versus vitamin A					
n	6	3	2	1	0
Estimate	4.7	5.6	0.54	0.51	–
95% CI	(1.5 to 7.8)	(−3.5 to 14.7)	(0.11 to 2.56)	(0.31 to 0.85)	–
Iron versus control/placebo					
n	6	3	2	1	0
Estimate	8.0	8.4	0.27	0.34	–
95% CI	(3.1 to 12.8)	(5.3 to 11.4)	(0.16 to 0.45)	(0.18 to 0.66)	–
P value for interaction	0.27	0.72	0.49	n/a	n/a

*Weighted mean difference.

†Pooled risk ratio.

### Safety outcomes

No statistically significant changes due to oral iron supplementation were observed for anthropometric or infectious indices (table 2). In meta-regression analyses, no significant differential impacts of iron on these outcomes were seen by schedule, duration or dose, except for an observed increase in the cumulative incidence of respiratory illness associated with iron supplements that were 1–2 per week, low dose or lasting for ≥7 months (online supplemental appendix 9). However, these differential effects were due to a single factorial trial of iron and polyunsaturated fatty acids that differed from the other trials in terms of schedule, dose and duration^22^; excluding this trial, no significant difference remained. Iron increased height-for-age z score (HAZ) by 0.20 (95% CI 0.01, 0.40) in trial arms composed exclusively of anaemic children (but based on only n=2 trial arms); no effect on HAZ was seen among trial arms of non-anaemic children (−0.02 (−0.18, 0.14); n=3) or mixed anaemic and non-anaemic children (0.00 (−0.03, 0.02); n=25) (p interaction=0.013). Improvements in cognitive development were seen in one trial arm among anaemic children (Bayley Cognitive SMD=1.39 (0.75, 2.04); Bayley Motor SMD=1.46 (0.81, 2.11)) compared with no effect seen in four trial arms among non-anaemic children (Bayley Cognitive SMD=0.29 (−0.12, 0.71); Bayley Motor SMD=0.03 (−0.31, 0.38)) or four trial arms among mixed anaemic and non-anaemic children (Bayley Cognitive SMD=0.07 (−0.10, 0.23); Bayley Motor SMD=0.11 (−0.16, 0.37)) (p interaction=0.020 for cognitive and 0.023 for motor scores).

### Risk of bias

The risk of bias within each trial is reported in online supplemental appendix 10. Many trials (16%–75%) did not report sufficient information to calculate the risk of bias according to one or more of the five criteria (online supplemental appendix 11). Forty-eight of the 129 trials (37%) were judged to be at high risk of bias for at least one of the criteria, with incomplete outcome data being the most frequent reason for a study to be assessed at high risk of bias (n=24, 19%). In sensitivity analyses excluding these 48 studies, the effects of iron supplementation on haematologic outcomes were similar (online supplemental appendix 12).

### Small study effects

For haematologic outcomes, studies with larger SEs tended to demonstrate more protective effect sizes (Egger’s test p<0.001) (online supplemental appendix 13). Attenuated but still statistically significant benefits were obtained during trim-and-fill sensitivity analyses for haemoglobin (2.2 g/L (1.2, 3.1)) and ferritin (4.1 ng/mL (1.6, 6.5)).

## Discussion

This systematic review and meta-analysis of 129 randomised controlled trials, including 201 trial arms of oral iron supplementation in children, demonstrates significant benefits on haematologic outcomes including haemoglobin (+6.3 g/L; 95% CI 5.5, 7.1), ferritin (+18.5 ng/mL; 16.1, 20.9) and prevalence of anaemia (39% reduction; 33%, 45%), ID (70% reduction; 63%, 76%) and IDA (80% reduction; 69%, 87%). Children under age 5 years were most frequently studied (63% of trial arms), including trials throughout this age range, but with meaningful numbers of trials among children aged 5–11 years (25%) and adolescents aged 12–19 years (12%). In sum, these findings provide strong evidence for the benefits of iron supplementation among children.

Importantly, the number and diversity of identified trials allowed us to assess factors that might modify these benefits. Our results suggest that frequent (3–7 times/week) and intermittent (1–2 times/week) iron supplementation may be equally effective at increasing haemoglobin and decreasing anaemia, ID and IDA. While WHO recommends daily oral iron supplementation for all children in regions with an anaemia prevalence of 40% or more, the success of such programmes may be threatened by low adherence from adverse gastrointestinal reactions or high caregiver burden to provide daily supplements.^23^ Weekly iron supplementation has been promoted as an alternative to reduce these barriers.^24 25^ Furthermore, since mammalian gastrointestinal epithelial cells turn over every 2–6 days, weekly supplementation may not be at a great disadvantage relative to daily supplementation with respect to the total amount of absorbed iron.^26 27^ Some evidence points to changes in gastrointestinal epithelial cells following a large bolus of iron that results in reduced transport of iron into portal blood.^28^ A prior meta-analysis of 21 trials concluded that, compared with daily iron supplements, intermittent supplementation had similar effects on haemoglobin levels but was less effective in reducing anaemia.^29^ Our findings, based on a much larger number of trials, suggest that frequent and intermittent supplementation are similarly effective in reducing anaemia.

Studies of longer duration were more likely to have a lower prevalence of baseline anaemia (online supplemental appendix 5). As a result, the apparently diminishing impacts of iron with increased duration were entirely explained on control for baseline anaemia (online supplemental appendix 6). Some prior studies have shown that impacts of iron on haemoglobin persist for several months after the cessation of supplementation,^30 31^ though the period of durability likely depends on the availability of dietary iron, burden of infection and degree of blood loss experienced by the population. The findings of this study support the current WHO recommendation of a 3-month course of iron supplementation, though a longer duration may be considered in order to maintain haemoglobin levels.

For all ages, the lowest dose tertile of iron received less than the WHO recommended daily supplement (10–12.5 mg/day for ages 6–23 months, 30 mg/day for ages 24–59 months, 30–60 mg/day for ages 5–12 years). Moderate age-adjusted doses appeared to be more effective than lower doses at increasing haemoglobin, but all doses effectively improved outcomes, even doses below current WHO recommendations. Interestingly, more frequent supplementation, longer durations of supplementation and higher doses each were associated with greater increases in serum ferritin. These novel findings can inform the design of future supplementation programmes, suggesting that flexibility is possible depending on specific aims.

Importantly, we found that benefits were generally similar across diverse ages from <5 months to >12 years, in males and in females and across world regions. This supports initiation of iron supplementation programmes in a diverse range of young populations. As might be expected, increases in haemoglobin and reductions in anaemia were approximately twice as large in populations in which all children were anaemic at baseline, compared with mixed or non-anaemic populations. Yet, even among children who were non-anaemic at baseline, iron supplementation effectively reduced the future risk of anaemia by 37%, and of ID and IDA by about 80%. These findings indicate that iron supplementation is effective for primary prevention among at-risk children.

Factorial trials of iron and zinc supplementation (n=11) found that cosupplementation of iron with zinc did not diminish impacts on haemoglobin, serum ferritin, ID or IDA. However, there was some evidence that iron supplementation alone decreases anaemia more than when given along with zinc supplementation. There was also a qualitative trend towards attenuated effects for all outcomes in children who received iron and zinc concurrently. Uptake of both iron and zinc is mediated by divalent metal transporter-1 and ferroportin, which may result in absorptive antagonism.^32 33^ However, the evidence from this meta-analysis suggests that in contexts where prevalent zinc deficiency is suspected, cosupplementation of iron can still yield population benefits.

There was no statistically significant difference in the effect of iron comparing children in factorial trials randomised additionally to vitamin A versus those randomised to no vitamin A (n=6 trials). The mechanism of iron and vitamin A interaction remains incompletely understood. Prior research has indicated that vitamin A may increase nonheme iron absorption, which is perhaps achieved through mobilising iron stores or stimulating the synthesis of transferrin.^34–36^ However, one bioavailability study in non-anaemic adults found that absorption of a single dose of iron (10 mg) was increased when cosupplemented with a dose of vitamin A of 1500 or 3000 IU, but inhibited when cosupplemented with dose of vitamin A of 6000 IU.^37^ Two trial arms in this meta-analysis gave single vitamin A doses of 200 000 IU at enrolment, two trial arms gave daily doses of 5000 or 10 000 IU and two trial arms gave 15 000 IU weekly. On balance, the evidence from this meta-analysis suggests that vitamin A can be given in combination with iron in order to improve haematologic outcomes.

In our meta-analysis, iron supplementation had no significant effect on infections, although small effects on diarrhoea or malaria could not be excluded. Iron is an essential nutrient for pathogenic microbes, and several studies have investigated whether iron status or supplementation relates to infection risk.^38^ One trial of iron-folic acid supplementation in a malaria-endemic region was halted early due to higher mortality among those randomised to iron^39^; and Cochrane review concluded that iron supplementation in malaria-endemic areas without malaria control programmes may increase the incidence of malaria, although iron may also reduce clinical malaria in regions where malaria control programmes are available.^10^ Relatively few studies included in this meta-analysis reported data on infections, those which did reported varying outcomes (eg, incidence, prevalence, mean number of episodes, average duration) and several studies excluded children on the basis of an infection, creating a possibility of reporting and selection bias. Our findings add to the body of literature on iron and infection, and the interaction between iron supplementation and infection remains an important area for future study.

Forty-three trials reported data on height-for-age, and no significant effects were seen on child growth. This finding is consistent with results of prior meta-analyses based on fewer studies.^9 11 12^ Furthermore, no effect modification was seen for growth-related outcomes for any of the eight potential modifiers that were investigated. Combined with the findings of prior research, this meta-analysis suggests that iron supplementation does not promote linear growth in children. With respect to cognitive function, a diverse set of measures and reporting scales was used; using the most frequently reported measure, the Bayley Index, we found some evidence of benefits of iron supplementation on mental performance (p=0.05).

Potential limitations of this research should be considered. Many studies had insufficient information that could be used to determine risk of bias, thereby impeding the exploration of iron effects according to study quality. Furthermore, when data were available on adherence to iron supplements, it was reported in numerous ways, similarly preventing a quantitative summary. As with all meta-analyses, publication bias cannot be ruled out; trim-and-fill methods suggested that effects were attenuated but still statistically significant when accounting for potentially missing studies. A large number of tests for effect heterogeneity were conducted (total of 25 tests across tables 3 and 4, and 110 in online supplemental appendices 7 and 8) without adjustment for multiple comparisons, and the positive findings from these tests should be interpreted accordingly with caution. Lastly, results for effect heterogeneity can be confounded due to collinear effect modifiers. Although we conduct sensitivity analyses controlling for baseline anaemia, the lack of individual-level data reduces the degree to which this and other factors can be precisely controlled.

The 2016 WHO guidelines on iron supplementation in infants and children were informed by systematic reviews of randomised controlled iron supplementation trials that found clear benefits of iron supplementation on haematologic outcomes.^9–12^ In this large systematic review of iron supplementation trials, our work extends these prior meta-analyses by exploring effect heterogeneity according to schedule, duration, dose and cosupplementation regimen. We find that the evidence supports the currently recommended dose and duration of iron supplementation, although weekly supplementation might be reasonable in certain contexts, especially at moderate or high doses. We also identified no evidence for harms of iron supplementation on anthropometrics or risk of infection, and possibly improved mental development. Furthermore, evidence from this meta-analysis suggests that cosupplementation of iron with zinc or vitamin A generally results in similar impacts on haematologic outcomes, although there is some evidence for these effects being attenuated for zinc. Our findings could be considered in clinical decision-making and the development of further guidelines on oral iron supplementation among children and adolescents.

## Data Availability

Data are available upon reasonable request.
