# Supplementary material for: Oral iron supplementation and anaemia in children according to schedule, duration, dose and cosupplementation: a systematic review and meta-analysis of 129 randomised trials
Source: BMJ Glob Health. 2023 Feb 27;8(2):e010745. doi: 10.1136/bmjgh-2022-010745 (PMC9972455; doi:10.1136/bmjgh-2022-010745)

**Appendices to "Oral iron supplementation and anemia in children according to schedule, duration, dose, and co-supplementation: a systematic review and meta-analysis of 129 randomized trials"**

**<u>Table of Contents</u>**

**Appendix 1:** Search terms. **2**

**Appendix 2:** Variables extracted from eligible studies. **3**

**Appendix 3.** Elemental iron supplementation dose tertile ranges. **7**

**Appendix 4.** References to included studies. **8**

**Appendix 5.** Characteristics of included studies. **16**

**Appendix 6.** Modification of iron effects after control for baseline anemia. **36**

**Appendix 7.** Effect modification of iron by baseline anemia, child age, child sex, WHO region, and iron formulation. **37**

**Appendix 8.** Comparison of iron supplementation effects within factorial trials, using double placebo as a common referent group. **40**

**Appendix 9.** Effect heterogeneity p-values for anthropometric, infectious, and development outcomes.*† **41**

**Appendix 10.** Risk of bias within included studies. **42**

**Appendix 11.** Cochrane risk of bias assessment of included studies (n=129). **45**

**Appendix 12.** Effect of oral iron supplementation versus placebo among children and adolescents aged <20 years among trials judged to not be at "high" risk of bias. **46**

**Appendix 13.** Assessment of small study bias for outcomes reported by ≥10 groups randomized to iron. **47**



Andersen CT, *et al. BMJ Global Health* 2023; 8:e010745. doi: 10.1136/bmjgh-2022-010745

**Appendix 1:** Search terms.

Title and abstract searches of the terms below were performed in PubMed, Scopus, Web of Science, and the Cochrane Central Register of Controlled Trials from inception through November 2020.

Infant OR Infan* OR newborn* OR new-born* OR perinat* OR neonat* OR child OR child OR child* OR children* OR schoolchild* OR schoolchild OR "school child" OR adolescent OR adolescen* OR youth* OR teen* OR pubescen* OR pediatrics OR pediatric* OR paediatric* OR peadiatric* OR school OR school* OR prematur* OR preterm* OR “Pregnant Women” OR Pregnancy OR pregnan* OR gravid OR obstetric OR antenatal OR antepartum OR gestation* OR lactation OR “breast feeding” OR lactation OR “breast feeding”[ Title/Abstract] OR breast-fe* OR breastfe* OR breast-milk OR breastmilk OR “lactating mother*” OR “lactating woman” OR “lactating women”

AND

iron OR hematinics OR “ferrous” OR “ferric” OR “hematinic” OR “haematinic” OR “haematinics” OR “iron compounds”

AND

“randomized controlled trial” OR “controlled clinical trial” OR “clinical trial” OR randomized OR randomised OR placebo OR random* OR trial

**Appendix 2:** Variables extracted from eligible studies.

| **Study characteristics** |
|---|
| Endnote Record Number |
| First author (last name) |
| Publication year |
| Corresponding author (full name) |
| Corresponding author (email address) |
| Study name |
| Country |
| Rural/Urban |
| Altitude for the study site (meters) |
| According to study report, is malaria endemic? |
| According to study report, are helminths endemic? |
| Is this a subgroup analysis of a priorly extracted study? |
| Which subgroup is being reported here? |
| Does this paper reference other publications using the same trial or data? If so, indicate the Endnote record numbers for those studies. |
| Notes on this paper |
| **Intervention characteristics** |
| Mode of supplementation |
| Dose of intervention (mg of elemental iron) |
| Dose of intervention (mg) per kg bodyweight |
| Mean body weight for the intervention group (kg). |
| Frequency of intervention |
| Unit of intervention frequency |
| Formulation of Iron |
| Total dose of supplement (used for conversion to elemental iron if elemental unavailable) |
| Unit for dose |
| Duration of the intervention (weeks) (there are 4.3 weeks per month) |
| Is outcome data post intervention endline available? |
| Year at baseline (if range pick median) |
| Were all children in the study dewormed as part of the study protocol? |
| What other compounds were given along with iron (to both intervention & control groups)? |
| Is this a factorial trial? |
| Daily dose consumed of other compound given. |
| Unit for daily dose of other compound given. |
| Did the control group receive a placebo? |
| Is the control group being compared to multiple intervention groups? |
| Population group targeted by intervention |
| List study inclusion and exclusion criteria |
| Is compliance reported in the paper? |
| Indicate the compliance data reported |
| Other notes on intervention |
| **Population characteristics** |
| Is population characteristic data given for the full sample or for the control group only? (Use the full sample data whenever possible.) |

Proportion of female children at baseline (%)
Child age at supplementation start/baseline (mean/median months)
Child age at baseline (SD)
Child age at baseline (min)
Child age at baseline (max)
Child age at outcome assessment (mean/median months)
Child age at outcome assessment (SD)
Age reported if other than age at supplementation start
Mean HAZ at baseline
Mean WHZ at baseline
Mean WAZ at baseline
Mean BAZ at baseline
Proportion of iron deficient children if reported (%)
Cutoff used to define iron deficiency
Proportion of anemic children if reported (%)
Cutoff used to define anemia (g/dl)
Proportion of children with iron deficiency anemia (%)
Cutoff used to define iron deficiency anemia
Proportion of children stunted at baseline (%)
Proportion of children low birth weight at baseline (%)
Cutoff used to define low birth weight (g)
Proportion of children born premature (%)
Cutoff used to define prematurity (wks)
Maternal age at baseline (mean/median years)
Maternal age at baseline (SD)
If pregnant, gestational age at baseline (mean/median weeks)
If pregnant, gestational age (sd)
If any family socioeconomic status measures are reported, please describe
Proportion of mothers who are iron deficient, if reported (%)
Proportion of mothers anemic at baseline, if reported (%)

**Outcome characteristics**

Specify outcome
Outcome unit
Method of Assessment
Extra information pertaining to outcome
Comparator group
How many weeks after baseline was the outcome measured?
How many participants were initially recruited for treatment?
How many participants were initially recruited for control?
Intervention total group sample size (total analyzed)
Was the difference-in-differences measure calculated in an adjusted regression model?
Difference in the differences between baseline & follow-up between intervention & control group (mean)
Difference in the differences between baseline & follow-up between intervention & control group (SD)
Difference in the differences between baseline & follow-up between intervention & control group (SE)

Difference in the differences between baseline & follow-up between intervention & control group (LCI)
Difference in the differences between baseline & follow-up between intervention & control group (UCI)
Difference in the differences between baseline & follow-up between intervention & control group (p-value)
Intervention group baseline sample size
Intervention group baseline estimate (mean)
Intervention group baseline estimate (SD)
Intervention group baseline estimate (SE)
Intervention group baseline estimate (LCI)
Intervention group baseline estimate (UCI)
Intervention group baseline estimate (LIQR)
Intervention group baseline estimate (UIQR)
Intervention group follow-up sample size
Intervention group follow-up estimate (mean)
Intervention group follow-up estimate (SD)
Intervention group follow-up estimate (SE)
Intervention group follow-up estimate (LCI)
Intervention group follow-up estimate (UCI)
Intervention group follow-up estimate (LIQR)
Intervention group follow-up estimate (UIQR)
Was the intervention group difference measure calculated in an adjusted regression model?
Intervention group difference sample size
Intervention group difference in outcome between baseline & follow-up (mean)
Intervention group difference in outcome between baseline & follow-up (SD)
Intervention group difference in outcome between baseline & follow-up (SE)
Intervention group difference in outcome between baseline & follow-up (LCI)
Intervention group difference in outcome between baseline & follow-up (UCI)
Intervention group difference in outcome between baseline & follow-up (LIQR)
Intervention group difference in outcome between baseline & follow-up (UIQR)
Intervention group difference in outcome between baseline & follow-up (p-value)
Control group baseline sample size
Control group baseline estimate (mean)
Control group baseline estimate (SD)
Control group baseline estimate (SE)
Control group baseline estimate (LCI)
Control group baseline estimate (UCI)
Control group baseline estimate (LIQR)
Control group baseline estimate (UIQR)
Control group follow-up sample size
Control group follow-up estimate (mean)
Control group follow-up estimate (SD)
Control group follow-up estimate (SE)
Control group follow-up estimate (LCI)
Control group follow-up estimate (UCI)
Control group follow-up estimate (LIQR)



Andersen CT, *et al. BMJ Global Health* 2023; 8:e010745. doi: 10.1136/bmjgh-2022-010745

| | |
|---|---|
| | Control group follow-up estimate (UIQR) |
| | Was the control group difference measure calculated in an adjusted regression model? |
| | Control group difference sample size |
| | Control group difference in outcome between baseline & follow-up (mean) |
| | Control group difference in outcome between baseline & follow-up (SD) |
| | Control group difference in outcome between baseline & follow-up (SE) |
| | Control group difference in outcome between baseline & follow-up (LCI) |
| | Control group difference in outcome between baseline & follow-up (UCI) |
| | Control group difference in outcome between baseline & follow-up (LIQR) |
| | Control group difference in outcome between baseline & follow-up (UIQR) |
| | Control group difference in outcome between baseline & follow-up (p-value) |
| | Flag indicating whether SD/SE was imputed during data entry |
| | Note other outcomes reported in the study but not extracted |
| **Subgroup data** | |
| | Was sub-group data on outcome presented by gender? |
| | Was sub-group data on outcome presented by age group? |
| | Was sub-group data on outcome presented by SES group? |
| | Was sub-group data on outcome presented by baseline anemia status? |
| | Was sub-group data on outcome presented by baseline iron status? |
| | Was sub-group data on outcome presented by baseline stunting/birthweight status? |
| | Other subgroup reported? (specify) |
| **Cochrane risk of bias assessment** | |
| | Method for generating randomization sequence |
| | How were individuals randomly assigned to treatment? |
| | Number of clusters randomized (total) |
| | Risk of bias due to randomization method? |
| | Method for concealing treatment allocation |
| | Risk of bias due to allocation concealment? |
| | Were participants blinded? |
| | Were supplementation providers blinded? |
| | Bias due to lack of participant or supplement provider blinding? |
| | Were outcome assessors blinded? |
| | Bias due to lack of outcome assessor blinding? |
| | Percent drop-out in intervention group |
| | Percent drop-out in control group |
| | Were reasons for withdrawals similar across treatment groups? |
| | Were reasons for withdrawals associated with other important covariates? |
| | If withdrawals were related to the outcome, describe the reason for withdrawal. |
| | Were appropriate methods used to impute missing data? |
| | Was an intention-to-treat analyses conducted (i.e. analyzed as randomized)? |
| | Was a per protocol analysis conducted? |
| | Risk of bias due to incomplete outcome data? |
| | Were data reported for all pre-specified outcomes? |
| | Were data reported for all pre-specified sub-group analyses |
| | Risk of bias due to selective outcome reporting? |

**Appendix 3.** Elemental iron supplementation dose tertile ranges.

| | Tertile | | |
|---|---|---|---|
| | Low | Middle | High |
| mg/week | | | |
| 0 - 5 mo | 18.2 – 50.0 | 51.5 – 70.0 | 79.8 - 99.4 |
| 6 - 23 mo | 20.0 – 70.0 | 87.5 – 87.5 | 105.0 – 462.0 |
| 24 - 59 mo | 20.0 – 100.0 | 114.0 – 207.9 | 210.0 – 420.0 |
| 5 - 11 y | 18.2 – 120.0 | 143.6 – 380.8 | 403.1 – 650.0 |
| 12 - 19 y | 50.0 – 100.0 | 120.0 – 250.0 | 300.0 – 1820.0 |
| | | | |
| mg/day | | | |
| 0 - 5 mo | 2.6 – 7.1 | 7.4 – 10.0 | 11.4 – 14.2 |
| 6 - 23 mo | 2.9 – 10.0 | 12.5 – 12.5 | 15.0 – 66.0 |
| 24 - 59 mo | 2.9 – 14.3 | 16.3 – 29.7 | 30.0 – 60.0 |
| 5 - 11 y | 2.6 – 17.1 | 20.5 – 54.4 | 57.6 – 92.9 |
| 12 - 19 y | 7.1 – 14.3 | 17.1 – 35.7 | 42.9 – 260.0 |

Note 1: The recommended dose of iron increases as children age. To account for the strong correlation between age and dose, we grouped children into five age categories and then categorized them into low, moderate, or high tertiles based on the total amount of iron they received each week.
Note 2: Tertiles reported here are for studies which reported hematologic outcomes and had sufficient data to calculate dose (n=94 iron treatment groups).

**Appendix 4.** References to included studies.

1. Adish AA, Esrey SA, Gyorkos TW, et al. Effect of consumption of food cooked in iron pots on iron status and growth of young children: a randomised trial. *Lancet (London, England)* 1999;353(9154):712-6. doi: 10.1016/s0140-6736(98)04450-x [published Online First: 1999/03/12]
2. Aggarwal D, Sachdev HP, Nagpal J, et al. Haematological effect of iron supplementation in breast fed term low birth weight infants. *Archives of disease in childhood* 2005;90(1):26-9. doi: 10.1136/adc.2003.040410 [published Online First: 2004/12/23]
3. Aguayo VM. School-administered weekly iron supplementation--effect on the growth and hemoglobin status of non-anemic Bolivian school-age children. A randomized placebo-controlled trial. *European journal of nutrition* 2000;39(6):263-9. [published Online First: 2001/06/09]
4. Akman M, Cebeci D, Okur V, et al. The effects of iron deficiency on infants' developmental test performance. *Acta paediatrica (Oslo, Norway : 1992)* 2004;93(10):1391-6. [published Online First: 2004/10/27]
5. Angeles IT, Schultink WJ, Matulessi P, et al. Decreased rate of stunting among anemic Indonesian preschool children through iron supplementation. *The American journal of clinical nutrition* 1993;58(3):339-42. [published Online First: 1993/09/01]
6. Angulo-Barroso RML, M.: Santos, D. C.: Bian, Y.: Sturza, J.: Jiang, Y.: Kaciroti, N.: Richards, B.: Lozoff, B. Iron Supplementation in Pregnancy or Infancy and Motor Development: A Randomized Controlled Trial. *Pediatrics* 2016;137(4) doi: 10.1542/peds.2015-3547 [published Online First: 2016/03/05]
7. Arcanjo FP, Arcanjo CC, Amancio OM, et al. Weekly iron supplementation for the prevention of anemia in pre-school children: a randomized, double-blind, placebo-controlled trial. *Journal of tropical pediatrics* 2011;57(6):433-8. doi: 10.1093/tropej/fmq119 [published Online First: 2011/02/03]
8. Arcanjo FPN, Santos PR, Arcanjo CPC, et al. Daily and Weekly Iron Supplementations are Effective in Increasing Hemoglobin and Reducing Anemia in Infants. *Journal of tropical pediatrics* 2013;59(3):175-79. doi: 10.1093/tropej/fms071
9. Aukett MA, Parks YA, Scott PH, et al. Treatment with iron increases weight gain and psychomotor development. *Archives of disease in childhood* 1986;61(9):849-57. [published Online First: 1986/09/01]
10. Ayoya MA, Spiekermann-Brouwer GM, Traore AK, et al. Multiple micronutrients including iron are not more effective than iron alone for improving hemoglobin and iron status of Malian school children. *The Journal of nutrition* 2009;139(10):1972-9. doi: 10.3945/jn.109.106625 [published Online First: 2009/08/14]
11. Ayoya MA, Spiekermann-Brouwer GM, Traore AK, et al. Effect on school attendance and performance of iron and multiple micronutrients as adjunct to drug treatment of Schistosoma-infected anemic schoolchildren. *Food and nutrition bulletin* 2012;33(4):235-41. [published Online First: 2013/02/22]
12. Ballin A, Berar M, Rubinstein U, et al. Iron state in female adolescents. *American journal of diseases of children (1960)* 1992;146(7):803-5. [published Online First: 1992/07/01]
13. Baqui AH, Zaman K, Persson LA, et al. Simultaneous weekly supplementation of iron and zinc is associated with lower morbidity due to diarrhea and acute lower respiratory infection in Bangladeshi infants. *The Journal of nutrition* 2003;133(12):4150-7. [published Online First: 2003/12/04]
14. Barclay SM, Aggett PJ, Lloyd DJ, et al. Reduced erythrocyte superoxide dismutase activity in low birth weight infants given iron supplements. *Pediatric research* 1991;29(3):297-301. doi: 10.1203/00006450-199103000-00015 [published Online First: 1991/03/01]
15. Baumgartner JS, C. M.: Malan, L.: Kvalsvig, J.: van Stuijvenberg, M. E.: Hurrell, R. F.: Zimmermann, M. B. Effects of iron and n-3 fatty acid supplementation, alone and in combination, on cognition in school children: a randomized, double-blind, placebo-controlled intervention in South Africa. *The American journal of clinical nutrition* 2012;96(6):1327-38. doi: 10.3945/ajcn.112.041004 [published Online First: 2012/10/26]
16. Berger J, Aguayo VM, Tellez W, et al. Weekly iron supplementation is as effective as 5 day per week iron supplementation in Bolivian school children living at high altitude. *European journal of clinical nutrition* 1997;51(6):381-6. [published Online First: 1997/06/01]
17. Berger J, Dyck JL, Galan P, et al. Effect of daily iron supplementation on iron status, cell-mediated immunity, and incidence of infections in 6-36 month old Togolese children. *European journal of clinical nutrition* 2000;54(1):29-35. [published Online First: 2000/03/01]

18. Berger J, Ninh NX, Khan NC, et al. Efficacy of combined iron and zinc supplementation on micronutrient status and growth in Vietnamese infants. *European journal of clinical nutrition* 2006;60(4):443-54. doi: 10.1038/sj.ejcn.1602336 [published Online First: 2005/11/25]
19. Berglund S, Westrup B, Domellof M. Iron supplements reduce the risk of iron deficiency anemia in marginally low birth weight infants. *Pediatrics* 2010;126(4):e874-83. doi: 10.1542/peds.2009-3624 [published Online First: 2010/09/08]
20. Bhatia D, Seshadri S. Growth performance in anemia and following iron supplementation. *Indian pediatrics* 1993;30(2):195-200. [published Online First: 1993/02/01]
21. Black MM, Baqui AH, Zaman K, et al. Iron and zinc supplementation promote motor development and exploratory behavior among Bangladeshi infants. *American Journal of Clinical Nutrition* 2004;80(4):903-10.
22. Bora R, Ramasamy S, Brown B, et al. Effect of iron supplementation from neonatal period on the iron status of6-month-old infants at-risk for early iron deficiency: a randomized interventional trial. *J Matern-Fetal Neonatal Med* 2019 doi: 10.1080/14767058.2019.1638358
23. Bruner AB, Joffe A, Duggan AK, et al. Randomised study of cognitive effects of iron supplementation in non-anaemic iron-deficient adolescent girls. *Lancet (London, England)* 1996;348(9033):992-96. doi: 10.1016/S0140-6736(96)02341-0
24. Burman D. Haemoglobin levels in normal infants aged 3 to 24 months, and the effect of iron. *Archives of disease in childhood* 1972;47(252):261-71. doi: 10.1136/adc.47.252.261
25. Buzina-Suboticanec KB, R.: Stavljenic, A.: Tadinac-Babic, M.: Juhovic-Markus, V. Effects of iron supplementation on iron nutrition status and cognitive functions in children. *Food and nutrition bulletin* 1998;19(4):298-306.
26. Charoenlarp P, Pholpothi T, Chatpunyaporn P, et al. The effect of riboflavin on the hematologic changes in iron supplementation of schoolchildren. *The Southeast Asian journal of tropical medicine and public health* 1980;11(1):97-103. [published Online First: 1980/03/01]
27. Chen KL, Y. F.: Chen, L.: Zhang, X.: Liu, Y. X.: Chen, J.: Li, T. Y. [Effects of vitamin A, vitamin A plus iron and multiple micronutrient-fortified seasoning powder on iron metabolic homeostasis]. *Zhonghua er ke za zhi Chinese journal of pediatrics* 2011; 49(12). http://onlinelibrary.wiley.com/o/cochrane/clcentral/articles/180/CN-00881180/frame.html.
28. Chen K, Chen XR, Zhang L, et al. Effect of simultaneous supplementation of vitamin A and iron on diarrheal and respiratory tract infection in preschool children in Chengdu City, China. *Nutrition (Burbank, Los Angeles County, Calif)* 2013;29(10):1197-203. doi: 10.1016/j.nut.2013.03.025
29. Chen K, Zhang L, Luo HY, et al. No enhancing effect of vitamin A administration on iron absorption or body total iron content in preschool children from Chengdu, China. *Journal of nutritional science and vitaminology* 2014;60(4):223-30. [published Online First: 2014/10/10]
30. Cheng XHP, H.: Wang, Y. B. [The Effect of Low Molecular Protein Iron on Iron Deficiency Anemia]. *Zhongguo shi yan xue ye xue za zhi / Zhongguo bing li sheng li xue hui = Journal of experimental hematology / Chinese Association of Pathophysiology* 2001;9(3):240-42. [published Online First: 2003/02/13]
31. Choe YH, Kim SK, Son BK, et al. Randomized placebo-controlled trial of Helicobacter pylori eradication for iron-deficiency anemia in preadolescent children and adolescents. *Helicobacter* 1999;4(2):135-9. [published Online First: 1999/06/26]
32. Chwang LC, Soemantri AG, Pollitt E. Iron supplementation and physical growth of rural Indonesian children. *The American journal of clinical nutrition* 1988;47(3):496-501. [published Online First: 1988/03/01]
33. Das BK, Bal MS, Tripathi AM, et al. Evaluation of frequency and dose of iron and other hematinics--an alternative strategy for anemia prophylaxis in rural preschoolers. *Indian pediatrics* 1984;21(12):933-8. [published Online First: 1984/12/01]
34. de Silva AA, S.: Weerasinghe, I.: Ahluwalia, N. Iron supplementation improves iron status and reduces morbidity in children with or without upper respiratory tract infections: a randomized controlled study in Colombo, Sri Lanka. *The American journal of clinical nutrition* 2003;77(1):234-41. [published Online First: 2002/12/25]
35. Desai MR, Mei JV, Kariuki SK, et al. Randomized, controlled trial of daily iron supplementation and intermittent sulfadoxine-pyrimethamine for the treatment of mild childhood anemia in western Kenya. *Journal of Infectious Diseases* 2003;187(4):658-66. doi: 10.1086/367986

36. Devaki PBC, R. K.: Geisser, P. Effects of oral supplementation with iron(III) hydroxide polymaltose complex on the hematological profile of adolescents with varying iron status. *Arzneimittel-Forschung* 2008;58(8):389-97. doi: 10.1055/s-0031-1296526 [published Online First: 2008/09/24]
37. Dewey KG, Domellof M, Cohen RJ, et al. Iron supplementation affects growth and morbidity of breast-fed infants: Results of a randomized trial in Sweden and Honduras. *Journal of Nutrition* 2002;132(11):3249-55.
38. Dijkhuizen MA, Wieringa FT, West CE, et al. Effects of iron and zinc supplementation in Indonesian infants on micronutrient status and growth. *The Journal of nutrition* 2001;131(11):2860-5. [published Online First: 2001/11/06]
39. Dijkhuizen MA, Winichagoon P, Wieringa FT, et al. Zinc supplementation improved length growth only in anemic infants in a multi-country trial of iron and zinc supplementation in South-East Asia. *The Journal of nutrition* 2008;138(10):1969-75. [published Online First: 2008/09/23]
40. Domellof M, Cohen RJ, Dewey KG, et al. Iron supplementation of breast-fed Honduran and Swedish infants from 4 to 9 months of age. *The Journal of pediatrics* 2001;138(5):679-87. doi: 10.1067/mpd.2001.112895 [published Online First: 2001/05/09]
41. Dossa RA, Ategbo EA, de Koning FL, et al. Impact of iron supplementation and deworming on growth performance in preschool Beninese children. *European journal of clinical nutrition* 2001;55(4):223-8. doi: 10.1038/sj.ejcn.1601126 [published Online First: 2001/05/22]
42. Dossa RAA, E. A.: Van Raaij, J. M.: de Graaf, C.: Hautvast, J. G. Multivitamin-multimineral and iron supplementation did not improve appetite of young stunted and anemic Beninese children. *The Journal of nutrition* 2001;131(11):2874-9. [published Online First: 2001/11/06]
43. Eftekhari MH, Simondon KB, Jalali M, et al. Effects of administration of iron, iodine and simultaneous iron-plus-iodine on the thyroid hormone profile in iron-deficient adolescent Iranian girls. *European journal of clinical nutrition* 2006;60(4):545-52. doi: 10.1038/sj.ejcn.1602349 [published Online First: 2005/12/13]
44. Elwood PCW, W. E.: Greene, W. J. W. EVALUATION OF IRON SUPPLEMENTS IN PREVENTION OF IRON-DEFICIENCY ANÆMIA. *The Lancet* 1970;296(7665):175-77. doi: 10.1016/S0140-6736(70)92534-1
45. Engstrom EM, Castro IR, Portela M, et al. Effectiveness of daily and weekly iron supplementation in the prevention of anemia in infants. *Revista de saude publica* 2008;42(5):786-95. [published Online First: 2008/08/19]
46. Ermis B, Demirel F, Demircan N, et al. Effects of three different iron supplementations in term healthy infants after 5 months of life. *Journal of tropical pediatrics* 2002;48(5):280-4. [published Online First: 2002/10/31]
47. Fahmida U, Rumawas JS, Utomo B, et al. Zinc-iron, but not zinc-alone supplementation, increased linear growth of stunted infants with low haemoglobin. *Asia Pacific journal of clinical nutrition* 2007;16(2):301-9. [published Online First: 2007/05/01]
48. Fallahi E, Kimiagar M, Nazari A, et al. Effect of zinc and iron supplementation on indicators of iron, zinc and vitamin A status of primary school children. *Pakistan journal of biological sciences : PJBS* 2007;10(7):1088-92. [published Online First: 2007/04/01]
49. Franz AR, Mihatsch WA, Sander S, et al. Prospective randomized trial of early versus late enteral iron supplementation in infants with a birth weight of less than 1301 grams. *Pediatrics* 2000;106(4):700-6. [published Online First: 2000/10/04]
50. Friel JK, Aziz K, Andrews WL, et al. A double-masked, randomized control trial of iron supplementation in early infancy in healthy term breast-fed infants. *The Journal of pediatrics* 2003;143(5):582-6. doi: 10.1067/s0022-3476(03)00301-9 [published Online First: 2003/11/15]
51. Fujiu T, Maruyama K, Koizumi T. Oral iron supplementation in preterm infants treated with erythropoietin. *Pediatrics international : official journal of the Japan Pediatric Society* 2004;46(6):635-9. doi: 10.1111/j.1442-200x.2004.01979.x [published Online First: 2005/01/22]
52. Gebreselassie H. Iron supplementation and malaria infection: results of a randomized controlled field trial. McGill University, 1996.
53. Geltman PL, Meyers AF, Bauchner H. Daily multivitamins with iron to prevent anemia in infancy: a randomized clinical trial. *Clinical pediatrics* 2001;40(10):549-54. [published Online First: 2001/10/30]
54. Geltman PL, Meyers AF, Mehta SD, et al. Daily multivitamins with iron to prevent anemia in high-risk infants: a randomized clinical trial. *Pediatrics* 2004;114(1):86-93. [published Online First: 2004/07/03]

55. Gokcay G, Ozden T, Karakas Z, et al. Effect of iron supplementation on development of iron deficiency anemia in breastfed infants. *Journal of tropical pediatrics* 2012;58(6):481-5. doi: 10.1093/tropej/fms028 [published Online First: 2012/07/04]
56. Gopaldas T, Kale M. Prophylactic iron supplementation for underprivileged school boys. I. Two levels of dosing and efficacy of teacher-distributions. *Indian pediatrics* 1985;22(10):731-6. [published Online First: 1985/10/01]
57. Gopaldas T, Kale M, Bhardwaj P. Prophylactic iron supplementation for underprivileged school boys. II. Impact on selected tests of cognitive function. *Indian pediatrics* 1985;22(10):737-43. [published Online First: 1985/10/01]
58. Greisen G. Mild anaemia in African school children: effect on running performance and an intervention trial. *Acta paediatrica Scandinavica* 1986;75(4):662-7. [published Online First: 1986/07/01]
59. Hacihamdioglu DOK, A. E.: Gursel, O.: Atay, A. A.: Balamtekin, N.: Aydin, A.: Hasimi, A.: Ozcan, O. Evaluation of lipid peroxidation and antioxidant system in healthy iron-replete infants receiving iron prophylaxis. *Nutrition (Burbank, Los Angeles County, Calif)* 2013;29(1):138-42. doi: 10.1016/j.nut.2012.05.009 [published Online First: 2012/11/03]
60. Harvey PWH, P. F.: Nesheim, M. C.: Galme, K.: Zegans, M.: Habicht, J. P.: Stephenson, L. S.: Radimer, K. L.: Brabin, B.: Forsyth, K.: et al.,. The effect of iron therapy on malarial infection in Papua New Guinean schoolchildren. *The American journal of tropical medicine and hygiene* 1989;40(1):12-8. [published Online First: 1989/01/01]
61. Hathirat P, Valyasevi A, Kotchabhakdi NJ, et al. Effects of an iron supplementation trial on the Fe status of Thai schoolchildren. *The British journal of nutrition* 1992;68(1):245-52. [published Online First: 1992/07/01]
62. Hess SY, Zimmermann MB, Adou P, et al. Treatment of iron deficiency in goitrous children improves the efficacy of iodized salt in Cote d'Ivoire. *The American journal of clinical nutrition* 2002; 75(4). http://onlinelibrary.wiley.com/o/cochrane/clcentral/articles/467/CN-00593467/frame.html.
63. Hettiarachchi M, Liyanage C, Wickremasinghe R, et al. The efficacy of micronutrient supplementation in reducing the prevalence of anaemia and deficiencies of zinc and iron among adolescents in Sri Lanka. *European journal of clinical nutrition* 2008;62(7):856-65. doi: 10.1038/sj.ejcn.1602791
64. Hieu NT, Sandalinas F, de Sesmaisons A, et al. Multi-micronutrient-fortified biscuits decreased the prevalence of anaemia and improved iron status, whereas weekly iron supplementation only improved iron status in Vietnamese school children. *The British journal of nutrition* 2012;108(8):1419-27. doi: 10.1017/s0007114511006945 [published Online First: 2012/01/17]
65. Hop LT, Berger J. Multiple micronutrient supplementation improves anemia, micronutrient nutrient status, and growth of Vietnamese infants: Double-blind, randomized, placebo-controlled trial. *Journal of Nutrition* 2005;135(3):660S-65S.
66. Htet MK, Fahmida U, Dillon D, et al. Is Iron Supplementation Influenced by Sub-Clinical Inflammation?: A Randomized Controlled Trial Among Adolescent Schoolgirls in Myanmar. *Nutrients* 2019;11(4) doi: 10.3390/nu11040918 [published Online First: 2019/04/27]
67. Idjradinata P, Pollitt E. Reversal of developmental delays in iron-deficient anaemic infants treated with iron. *Lancet (London, England)* 1993;341(8836):1-4. [published Online First: 1993/01/02]
68. Idjradinata P, Watkins WE, Pollitt E. Adverse effect of iron supplementation on weight gain of iron-replete young children. *Lancet (London, England)* 1994;343(8908):1252-4. [published Online First: 1994/05/21]
69. Irigoyen M, Davidson LL, Carriero D, et al. Randomized, placebo-controlled trial of iron supplementation in infants with low hemoglobin levels fed iron-fortified formula. *Pediatrics* 1991;88(2):320-6. [published Online First: 1991/08/01]
70. Jalambo M, Karim N, Naser I, et al. Effects of iron supplementation and nutrition education on haemoglobin, ferritin and oxidative stress in iron-deficient female adolescents in Palestine: randomized control trial. *East Mediterr Health J* 2018;24(6):560-68. doi: 10.26719/2018.24.6.560 [published Online First: 2018/08/07]
71. Kapur D, Sharma S, Agarwal KN. Effectiveness of nutrition education, iron supplementation or both on iron status in children. *Indian pediatrics* 2003;40(12):1131-44. [published Online First: 2004/01/15]
72. Kashyap PG, T. Hematinic supplementation and hematological status of underprivileged school girls (8-15 YRS of age). *Nutrition Research* 1987;7(11):1127-38. doi: 10.1016/S0271-5317(87)80038-6
73. Kashyap P, Gopaldas T. Impact of hematinic supplementation on cognitive function in underprivileged school girls (8-15 Yrs of age). *Nutrition Research* 1987;7(11):1117-26. doi: 10.1016/S0271-5317(87)80037-4
74. Kianfar H, Kimiagar M, Ghaffarpour M. Effect of daily and intermittent iron supplementation on iron status of high school girls. *International journal for vitamin and nutrition research Internationale Zeitschrift fur Vitamin- und*

*Ernahrungsforschung Journal international de vitaminologie et de nutrition* 2000;70(4):172-7. doi: 10.1024/0300-9831.70.4.172 [published Online First: 2000/09/16]
75. Kordas K, Stoltzfus RJ, Lopez P, et al. Iron and zinc supplementation does not improve parent or teacher ratings of behavior in first grade Mexican children exposed to lead. *The Journal of pediatrics* 2005;147(5):632-9. doi: 10.1016/j.jpeds.2005.06.037 [published Online First: 2005/11/18]
76. Kusumastuti AC, Ardiaria M, Hendrianingtyas M. Effect of Zinc and Iron Supplementation on Appetite, Nutritional Status and Intelligence Quotient in Young Children. *Indonesian Biomedical Journal* 2018;10(2):133-39. doi: 10.18585/inabj.v10i2.365
77. Lambert AK, K.: Scragg, R.: Metcalf, P.: Schaaf, D. Effects of iron treatment on cognitive performance and working memory in non-anaemic, iron-deficient girls. *New Zealand Journal of Psychology* 2002;31(1):19-28.
78. Latham MC, Stephenson LS, Kinoti SN, et al. Improvements in growth following iron supplementation in young Kenyan school children. *Nutrition (Burbank, Los Angeles County, Calif)* 1990;6(2):159-65. [published Online First: 1990/03/01]
79. Lawless JW, Latham MC, Stephenson LS, et al. Iron supplementation improves appetite and growth in anemic Kenyan primary school children. *The Journal of nutrition* 1994;124(5):645-54. [published Online First: 1994/05/01]
80. Lee JWP, C. K. Effect of Vitamin C Supplementation on Iron Status of Adolescent Girls with Low Hemoglobin Levels. *Korean Journal of Community Nutrition* 1997; 2(5). http://onlinelibrary.wiley.com/o/cochrane/clcentral/articles/827/CN-01045827/frame.html.
81. Leenstra T, Kariuki SK, Kurtis JD, et al. The effect of weekly iron and vitamin A supplementation on hemoglobin levels and iron status in adolescent schoolgirls in western Kenya. *European journal of clinical nutrition* 2009;63(2):173-82. doi: 10.1038/sj.ejcn.1602919 [published Online First: 2007/10/12]
82. Lind T, Lonnerdal B, Stenlund H, et al. A community-based randomized controlled trial of iron and zinc supplementation in Indonesian infants: interactions between iron and zinc. *American Journal of Clinical Nutrition* 2003;77(4):883-90.
83. Lind TL, B.: Stenlund, H.: Gamayanti, I. L.: Ismail, D.: Seswandhana, R.: Persson, L. A. A community-based randomized controlled trial of iron and zinc supplementation in Indonesian infants: effects on growth and development. *The American journal of clinical nutrition* 2004;80(3):729-36. [published Online First: 2004/08/24]
84. Loria ASM, L.: Arroyo, P. Nutritional anemia. VIII. Hemoglobin and plasma iron in infants treated prenatally with iron. *Nutrition Reports International* 1979;19(4):451-61.
85. Lozoff BJ, Y.: Li, X.: Zhou, M.: Richards, B.: Xu, G.: Clark, K. M.: Liang, F.: Kaciroti, N.: Zhao, G.: Santos, D. C.: Zhang, Z.: Tardif, T.: Li, M. Low-Dose Iron Supplementation in Infancy Modestly Increases Infant Iron Status at 9 Mo without Decreasing Growth or Increasing Illness in a Randomized Clinical Trial in Rural China. *The Journal of nutrition* 2016;146(3):612-21. doi: 10.3945/jn.115.223917 [published Online First: 2016/01/23]
86. Lopez de Romana GC, S.: Lopez de Romana, D.: Gross, R. Efficacy of multiple micronutrient supplementation for improving anemia, micronutrient status, growth, and morbidity of Peruvian infants. *The Journal of nutrition* 2005;135(3):646s-52s. [published Online First: 2005/03/01]
87. Majumdar I, Paul P, Talib VH, et al. The effect of iron therapy on the growth of iron-replete and iron-deplete children. *Journal of tropical pediatrics* 2003;49(2):84-8. [published Online First: 2003/05/06]
88. Malan L, Baumgartner J, Calder PC, et al. n-3 Long-chain PUFAs reduce respiratory morbidity caused by iron supplementation in iron-deficient South African schoolchildren: a randomized, double-blind, placebo-controlled intervention. *American Journal of Clinical Nutrition* 2015;101(3):668-79. doi: 10.3945/ajcn.113.081208
89. Massaga JJ, Kitua AY, Lemnge MM, et al. Effect of intermittent treatment with amodiaquine on anaemia and malarial fevers in infants in Tanzania: a randomised placebo-controlled trial. *Lancet (London, England)* 2003; 361(9372). http://onlinelibrary.wiley.com/o/cochrane/clcentral/articles/073/CN-00438073/frame.html.
90. Mebrahtu T, Stoltzfus RJ, Chwaya HM, et al. Low-dose daily iron supplementation for 12 months does not increase the prevalence of malarial infection or density of parasites in young Zanzibari children. *Journal of Nutrition* 2004;134(11):3037-41.
91. Mejia LA, Chew F. Hematological effect of supplementing anemic children with vitamin A alone and in combination with iron. *The American journal of clinical nutrition* 1988;48(3):595-600. [published Online First: 1988/09/01]
92. Menendez C, Kahigwa E, Hirt R, et al. Randomised placebo-controlled trial of iron supplementation and malaria chemoprophylaxis for prevention of severe anaemia and malaria in Tanzanian infants. *Lancet (London, England)* 1997;350(9081):844-50. doi: 10.1016/s0140-6736(97)04229-3 [published Online First: 1997/10/06]

93. Menendez C, Schellenberg D, Quinto L, et al. The effects of short-term iron supplementation on iron status in infants in malaria-endemic areas. *American Journal of Tropical Medicine and Hygiene* 2004;71(4):434-40.
94. Metallinos-Katsaras E, Valassi-Adam E, Dewey KG, et al. Effect of iron supplementation on cognition in Greek preschoolers. *European journal of clinical nutrition* 2004; 58(11). http://onlinelibrary.wiley.com/o/cochrane/clcentral/articles/089/CN-00505089/frame.html.
95. Mitra AK, Akramuzzaman SM, Fuchs GJ, et al. Long-term oral supplementation with iron is not harmful for young children in a poor community of Bangladesh. *Journal of Nutrition* 1997;127(8):1451-55.
96. Mozaffari-Khosravi H, Noori-Shadkam M, Fatehi F, et al. Once weekly low-dose iron supplementation effectively improved iron status in adolescent girls. *Biological trace element research* 2010;135(1-3):22-30. doi: 10.1007/s12011-009-8480-0 [published Online First: 2009/08/05]
97. Mwanri L, Worsley A, Ryan P, et al. Supplemental vitamin A improves anemia and growth in anemic school children in Tanzania. *The Journal of nutrition* 2000;130(11):2691-6. [published Online First: 2000/10/29]
98. Nagpal J, Sachdev HP, Singh T, et al. A randomized placebo-controlled trial of iron supplementation in breastfed young infants initiated on complementary feeding: effect on haematological status. *Journal of health, population, and nutrition* 2004;22(2):203-11. [published Online First: 2004/10/12]
99. Nair VKMB, A. S. Effect of Iron Prophylaxis in Preventing Iron Deficiency Anaemia in Term Infants. *Journal of Evolution of Medical and Dental Sciences-Jemds* 2017;6(7):559-61. doi: 10.14260/jemds/2017/119
100. Nchito M, Geissler PW, Mubila L, et al. The effect of iron and multi-micronutrient supplementation on Ascaris lumbricoides reinfection among Zambian schoolchildren. *Transactions of the Royal Society of Tropical Medicine and Hygiene* 2009;103(3):229-36. doi: 10.1016/j.trstmh.2008.08.005 [published Online First: 2008/10/22]
101. Northrop-Clewes CA, Paracha PI, McLoone UJ, et al. Effect of improved vitamin A status on response to iron supplementation in Pakistani infants. *The American journal of clinical nutrition* 1996;64(5):694-9. [published Online First: 1996/11/01]
102. Olsen A, Nawiri J, Friis H. The impact of iron supplementation on reinfection with intestinal helminths and Schistosoma mansoni in western Kenya. *Transactions of the Royal Society of Tropical Medicine and Hygiene* 2000;94(5):493-9. [published Online First: 2001/01/02]
103. Olsen A, Nawiri J, Magnussen P, et al. Failure of twice-weekly iron supplementation to increase blood haemoglobin and serum ferritin concentrations: results of a randomized controlled trial. *Annals of tropical medicine and parasitology* 2006;100(3):251-63. doi: 10.1179/136485906x91486 [published Online First: 2006/04/25]
104. Paganini D, Uyoga MA, Kortman GAM, et al. Prebiotic galacto-oligosaccharides mitigate the adverse effects of iron fortification on the gut microbiome: a randomised controlled study in Kenyan infants. *Gut* 2017;66(11):1956-67. doi: 10.1136/gutjnl-2017-314418 [published Online First: 2017/08/05]
105. Palupi L, Schultink W, Achadi E, et al. Effective community intervention to improve hemoglobin status in preschoolers receiving once-weekly iron supplementation. *The American journal of clinical nutrition* 1997;65(4):1057-61. [published Online First: 1997/04/01]
106. Paracha PIA, I.: Nawab, G. Effect of iron supplementation on biochemical indices of iron status in selected pre-adolescent schoolgirls in North West Frontier Province, Pakistan. *Asia Pacific journal of clinical nutrition* 1993;2(4):177-81. [published Online First: 1993/12/01]
107. Perrone L, Salerno M, Gialanella G, et al. Long-term zinc and iron supplementation in children of short stature: effect of growth and on trace element content in tissues. *Journal of trace elements in medicine and biology : organ of the Society for Minerals and Trace Elements (GMS)* 1999;13(1-2):51-6. doi: 10.1016/s0946-672x(99)80023-6 [published Online First: 1999/08/13]
108. Prasetyani MAdM, Q.: Afeworki, R.: Kaisar, M. M.: Stefanie, D.: Sartono, E.: Supali, T.: van der Ven, A. J. Effect of a short course of iron polymaltose on acquisition of malarial parasitaemia in anaemic Indonesian schoolchildren: a randomized trial. *Malaria journal* 2017;16(1):50. doi: 10.1186/s12936-017-1691-5 [published Online First: 2017/01/29]
109. Reeves JD, Yip R. Lack of adverse side effects of oral ferrous sulfate therapy in 1-year-old infants. *Pediatrics* 1985;75(2):352-5. [published Online First: 1985/02/01]
110. Rezaeian A, Ghayour-Mobarhan M, Mazloum SR, et al. Effects of iron supplementation twice a week on attention score and haematologic measures in female high school students. *Singapore medical journal* 2014;55(11):587-92. [published Online First: 2015/01/30]

111. Richard SA, Zavaleta N, Caulfield LE, et al. Zinc and iron supplementation and malaria, diarrhea, and respiratory infections in children in the Peruvian Amazon. *The American journal of tropical medicine and hygiene* 2006;75(1):126-32. [published Online First: 2006/07/14]
112. Rosado JL, Lopez P, Munoz E, et al. Zinc supplementation reduced morbidity, but neither zinc nor iron supplementation affected growth or body composition of Mexican preschoolers. *American Journal of Clinical Nutrition* 1997;65(1):13-19.
113. Rosado JL, Lopez P, Kordas K, et al. Iron and/or zinc supplementation did not reduce blood lead concentrations in children in a randomized, placebo-controlled trial. *The Journal of nutrition* 2006;136(9):2378-83. [published Online First: 2006/08/22]
114. Roschnik N, Parawan A, Baylon MA, et al. Weekly iron supplements given by teachers sustain the haemoglobin concentration of schoolchildren in the Philippines. *Tropical medicine & international health : TM & IH* 2004;9(8):904-9. doi: 10.1111/j.1365-3156.2004.01279.x [published Online First: 2004/08/12]
115. Sarker SA, Mahmud H, Davidsson L, et al. Causal relationship of Helicobacter pylori with iron-deficiency anemia or failure of iron supplementation in children. *Gastroenterology* 2008;135(5):1534-42. doi: 10.1053/j.gastro.2008.07.030 [published Online First: 2008/09/09]
116. Seshadri SG, T. Impact of iron supplementation on cognitive functions in preschool and school-aged children: the Indian experience. *The American journal of clinical nutrition* 1989;50(3 Suppl):675-84; discussion 85-6. [published Online First: 1989/09/01]
117. Smith AW, Hendrickse RG, Harrison C, et al. The effects on malaria of treatment of iron-deficiency anaemia with oral iron in Gambian children. *Annals of tropical paediatrics* 1989;9(1):17-23. [published Online First: 1989/03/01]
118. Smuts CM, Dhansay MA, Faber M, et al. Efficacy of multiple micronutrient supplementation for improving anemia, micronutrient status, and growth in south African infants. *Journal of Nutrition* 2005;135(3):653S-59S.
119. Smuts CMG, J.: Kvalsvig, J.: Zimmermann, M. B.: Baumgartner, J. Long-chain n-3 PUFA supplementation decreases physical activity during class time in iron-deficient South African school children. *The British journal of nutrition* 2014:1-13. doi: 10.1017/s0007114514003493 [published Online First: 2014/12/05]
120. Soemantri AG. Preliminary findings on iron supplementation and learning achievement of rural Indonesian children. *The American journal of clinical nutrition* 1989;50(3 Suppl):698-701; discussion 01-2. [published Online First: 1989/09/01]
121. Soewondo S, Husaini M, Pollitt E. Effects of iron deficiency on attention and learning processes in preschool children: Bandung, Indonesia. *The American journal of clinical nutrition* 1989;50(3 Suppl):667-73; discussion 73-4. [published Online First: 1989/09/01]
122. Stoltzfus RJ, Kvalsvig JD, Chwaya HM, et al. Effects of iron supplementation and anthelmintic treatment on motor and language development of preschool children in Zanzibar: double blind, placebo controlled study. *BMJ (Clinical research ed)* 2001;323(7326):1389-93. [published Online First: 2001/12/18]
123. Stoltzfus RJ, Chway HM, Montresor A, et al. Low dose daily iron supplementation improves iron status and appetite but not anemia, whereas quarterly anthelminthic treatment improves growth, appetite and anemia in Zanzibari preschool children. *The Journal of nutrition* 2004;134(2):348-56. [published Online First: 2004/01/30]
124. Sungthong R, Mo-Suwan L, Chongsuvivatwong V, et al. Once weekly is superior to daily iron supplementation on height gain but not on hematological improvement among schoolchildren in Thailand. *The Journal of nutrition* 2002;132(3):418-22. [published Online First: 2002/03/07]
125. Sungthong R, Mo-suwan L, Chongsuvivatwong V, et al. Once-weekly and 5-days a week iron supplementation differentially affect cognitive function but not school performance in Thai children. *The Journal of nutrition* 2004;134(9):2349-54. [published Online First: 2004/08/31]
126. Teshome EM, Andang'o PEA, Osoti V, et al. Daily home fortification with iron as ferrous fumarate versus NaFeEDTA: a randomised, placebo-controlled, non-inferiority trial in Kenyan children. *Bmc Medicine* 2017;15 doi: 10.1186/s12916-017-0839-z
127. Thibault H, Galan P, Selz F, et al. The immune response in iron-deficient young children: effect of iron supplementation on cell-mediated immunity. *European journal of pediatrics* 1993;152(2):120-4. [published Online First: 1993/02/01]

128. Untoro J, Karyadi E, Wibowo L, et al. Multiple micronutrient supplements improve micronutrient status and anemia but not growth and morbidity of Indonesian infants: a randomized, double-blind, placebo-controlled trial. *The Journal of nutrition* 2005;135(3):639s-45s. [published Online First: 2005/03/01]
129. van den Hombergh JD, E.: Smit, Y. Does iron therapy benefit children with severe malaria-associated anaemia? A clinical trial with 12 weeks supplementation of oral iron in young children from the Turiani Division, Tanzania. *Journal of tropical pediatrics* 1996;42(4):220-7. [published Online First: 1996/08/01]
130. Verhoef H, West CE, Nzyuko SM, et al. Intermittent administration of iron and sulfadoxine-pyrimethamine to control anaemia in Kenyan children: a randomised controlled trial. *Lancet (London, England)* 2002;360(9337):908-14. doi: 10.1016/S0140-6736(02)11027-0
131. Wang YJW, Q.: Yang, L. C.: Zhang, X. R.: Zeng, C. M.: Yang, X. G.: Liu, J. [The influence of preventive iron supplementation to iron nutritional status in breastfed infants]. [Chinese]. *Zhonghua yu fang yi xue za zhi [Chinese journal of preventive medicine]* 2012; 46(4). http://onlinelibrary.wiley.com/o/cochrane/clcentral/articles/710/CN-00920710/frame.html.
132. Wasantwisut E, Winichagoon P, Chitchumroonchokchai C, et al. Iron and zinc supplementation improved iron and zinc status, but not physical growth, of apparently healthy, breast-fed infants in rural communities of northeast Thailand. *The Journal of nutrition* 2006;136(9):2405-11. [published Online First: 2006/08/22]
133. Wieringa FT, Berger J, Dijkhuizen MA, et al. Combined iron and zinc supplementation in infants improved iron and zinc status, but interactions reduced efficacy in a multicountry trial in southeast Asia. *Journal of Nutrition* 2007;137(2):466-71.
134. Yalçin S, Yurdakök K, Açikgöz D, et al. Short-term developmental outcome of iron prophylaxis in infants. *Pediatrics International* 2000;42(6):625-30. doi: 10.1046/j.1442-200X.2000.01299.x
135. Yip RR, J. D.: Lonnerdal, B.: Keen, C. L.: Dallman, P. R. Does iron supplementation compromise zinc nutrition in healthy infants? *The American journal of clinical nutrition* 1985;42(4):683-7. [published Online First: 1985/10/01]
136. Yurdakök K, Temiz F, Yalçin SS, et al. Efficacy of Daily and Weekly Iron Supplementation on Iron Status in Exclusively Breast-Fed Infants. *Journal of pediatric hematology/oncology* 2004;26(5):284-88. doi: 10.1097/00043426-200405000-00005
137. Zavaleta N, Respicio G, Garcia T. Efficacy and acceptability of two iron supplementation schedules in adolescent school girls in Lima, Peru. *Journal of Nutrition* 2000;130(2):462S-64S.
138. Zhao X, Lu Q, Wang S, et al. [Efficiency of NaFeEDTA fortified soy sauce on anemia prevention]. *Wei sheng yan jiu = Journal of hygiene research* 2004;33(2):202-4. [published Online First: 2004/06/24]
139. Ziegler EE, Nelson SE, Jeter JM. Iron status of breastfed infants is improved equally by medicinal iron and iron-fortified cereal. *American Journal of Clinical Nutrition* 2009;90(1):76-87. doi: 10.3945/ajcn.2008.27350
140. Ziegler EE, Nelson SE, Jeter JM. Iron supplementation of breastfed infants from an early age. *The American journal of clinical nutrition* 2009;89(2):525-32. doi: 10.3945/ajcn.2008.26591 [published Online First: 2008/12/17]
141. Zlotkin SA, K. Y.: Schauer, C.: Yeung, G. Use of microencapsulated iron(II) fumarate sprinkles to prevent recurrence of anaemia in infants and young children at high risk. *Bulletin of the World Health Organization* 2003; 81(2). http://onlinelibrary.wiley.com/o/cochrane/clcentral/articles/518/CN-00437518/frame.html: http://www.ncbi.nlm.nih.gov/pmc/articles/PMC2572396/pdf/12756979.pdf.
142. Zlotkin S, Newton S, Aimone AM, et al. Effect of iron fortification on malaria incidence in infants and young children in Ghana: a randomized trial. *Jama* 2013;310(9):938-47. doi: 10.1001/jama.2013.277129 [published Online First: 2013/09/05]

**Appendix 5.** Characteristics of included studies.

| Author & Year | Country | Age | Intervention | Comparison group | Sample size | Outcomes |
|---|---|---|---|---|---|---|
| Adish, 1997 | Ethiopia | 24 mo - 5 yr | 30 mg elemental Fe as ferrous sulfate daily for 3 months | Placebo; or Placebo + Vitamin A (factorial trial) | n=399 total participants analyzed; n=196 treatment, n=203 control | Hemoglobin, Diarrhea, Respiratory infection |
| Aggarwal et al, 2005 | India | 50 days - 80 days | 3 mg/kg elemental Fe as ferric ammonium citrate daily for 8 weeks | Placebo | n=26 total participants analyzed; n=13 treatment, n=13 control | Hemoglobin, Ferritin |
| Aguayo et al, 2000 | Bolivia | 6 yr - 12 yr | 3 mg/kg elemental Fe as ferrous sulfate 1 time per week for 18 weeks | Placebo | n=64 total participants analyzed; n=33 treatment, n=31 control | Hemoglobin, Anemia, HAZ, WAZ |
| Akman et al, 2004 | Turkey | 6 mo - 30 mo | 6 mg/kg elemental Fe as ferrous glisine-sulphate daily for 3 months | Control | n=40 total participants analyzed; n=21 treatment, n=19 control | Hemoglobin, Ferritin |
| Angeles et al, 1993 | Indonesia | 24 mo - 6 yr | 30 mg elemental Fe as ferrous sulfate + Vitamin C daily for 2 months | Placebo + Vitamin C | n=76 total participants analyzed; n=39 treatment, n=37 control | Hemoglobin, Ferritin, HAZ, WHZ, WAZ, Diarrhea, Respiratory infection |
| Angulo-Barro et al, 2016 | China | ~1 mo (mean age) | 1 mg/kg elemental Fe as iron protein succinylate daily for 7.5 months | Placebo; or Placebo + Maternal iron (factorial trial) | n=1196 total participants analyzed; n=610 treatment, n=586 control | Cognitive |
| Arcanjo et al, 2011 | Brazil | ~5 yr (mean age) | 50 mg elemental Fe as ferrous sulfate 1 time per week for 14 weeks | Placebo | n=99 total participants analyzed; n=50 treatment, n=49 control | Hemoglobin, Anemia |
| Arcanjo et al, 2013 | Brazil | 12 mo - 24 mo | 25 mg elemental iron (formulation not specified) 1 time per week for 4 months; 12.5 mg elemental iron (formulation not specified) daily for 4 months | Placebo | n=176 total participants analyzed; n=120 treatment, n=56 control | Hemoglobin, Anemia |

| Author & Year | Country | Age | Intervention | Comparison group | Sample size | Outcomes |
|---|---|---|---|---|---|---|
| Aukett et al, 1986 | United Kingdom | 17 mo - 19 mo | 24 mg elemental Fe as ferrous sulfate + Vitamin C daily for 2 months | Placebo + Vitamin C | n=97 total participants analyzed; n=48 treatment, n=49 control | Hemoglobin, Ferritin, Anemia, Iron deficiency |
| Ayoya et al, 2009 | Mali | 7 yr - 13 yr | 60 mg elemental Fe as ferrous sulfate + Praziquantal 5 times per week for 12 weeks | Control + Praziquantal | n=202 total participants analyzed; n=105 treatment, n=97 control | Hemoglobin, Ferritin, Anemia, Iron deficiency anemia, Malaria |
| Ayoya et al, 2012 | Mali | 7 yr - 13 yr | 60 mg elemental Fe as ferrous sulfate + Praziquantal 5 times per week for 12 weeks | Control + Praziquantal | n=202 total participants analyzed; n=105 treatment, n=97 control | Cognitive |
| Ballin et al, 1992 | Israel | 16 yr - 18 yr | 105 mg elemental Fe as iron polystyrene sulfonate adsorbate daily for 2 months | Placebo | n=59 total participants analyzed; n=29 treatment, n=30 control | Iron deficiency |
| Baqui et al, 2003 | Bangladesh | ~6 mo (mean age) | 20 mg elemental Fe as ferrous sulfate 1 time per week for 6 months | Placebo + Riboflavin; or Placebo + Zinc + Riboflavin (factorial trial) | n=249 total participants analyzed; n=125 treatment, n=124 control | Hemoglobin, Ferritin, Diarrhea, Respiratory infection |
| Barclay et al, 1991 | United Kingdom | ~1 mo (mean age) | 13.8 mg elemental Fe as NaFeEDTA daily for 16 weeks; 7 mg elemental Fe as NaFeEDTA daily for 16 weeks | Control + Mutivitamin | n=55 total participants analyzed; n=36 treatment, n=19 control | Hemoglobin, Ferritin |
| Baumgartner et al, 2012 | South Africa | 6 yr - 12 yr | 50 mg elemental Fe as ferrous sulfate 4 times per week for 8.5 months | Placebo + Vitamin C; or Placebo + n-3 fatty acids + Vitamin C (factorial trial) | n=294 total participants analyzed; n=145 treatment, n=149 control | Hemoglobin, Ferritin, HAZ, WHZ, WAZ |



Andersen CT, *et al. BMJ Global Health* 2023; 8:e010745. doi: 10.1136/bmjgh-2022-010745

| **Author & Year** | **Country** | **Age** | **Intervention** | **Comparison group** | **Sample size** | **Outcomes** |
|---|---|---|---|---|---|---|
| Berger et al, 1997 | Bolivia | 3 yr - 8 yr | 3-4 mg/kg elemental Fe as ferrous sulfate 1 time per week for 16 weeks; 3-4 mg/kg elemental Fe as ferrous sulfate 5 times per week for 16 weeks | Placebo | n=173 total participants analyzed; n=116 treatment, n=57 control | Hemoglobin, Anemia |
| Berger et al, 2000 | Togo | 6 mo - 3 yr | 2-3 mg/kg elemental Fe as iron betainate daily for 3 months | Placebo | n=163 total participants analyzed; n=84 treatment, n=79 control | Hemoglobin, Ferritin, Anemia, Iron deficiency, Diarrhea, Respiratory infection, Malaria |
| Berger et al, 2006 | Vietnam | 4 mo - 7 mo | 10 mg elemental Fe as ferrous sulfate 7 times per week for 6 months | Placebo + Vitamin A; or Placebo + Vitamin A + Zinc (factorial trial) | n=770 total participants analyzed; n=384 treatment, n=386 control | Hemoglobin, Ferritin, Anemia, Iron deficiency, Iron deficiency anemia, HAZ, WHZ, WAZ, Stunting, Wasting, Diarrhea, Respiratory infection |
| Berglund et al, 2010 | Sweden | ~1 mo (mean age) | 1 mg/kg elemental Fe as ferrous succinate daily for 4.5 months; 2 mg/kg elemental Fe as ferrous succinate daily for 4.5 months | Placebo | n=243 total participants analyzed; n=160 treatment, n=83 control | Hemoglobin, Ferritin, Anemia, Iron deficiency, Iron deficiency anemia |
| Bhatia et al, 1993 | India | ~4 yr (mean age) | 40 mg elemental iron (formulation not specified) daily for 6 months | Placebo | n=156 total participants analyzed; n=84 treatment, n=72 control | Hemoglobin |
| Black et al, 2004 | Bangladesh | ~7 mo (mean age) | 20 mg elemental Fe as ferrous sulfate 1 time per week for 6 months | Placebo + Riboflavin + Vitamin A; or Placebo + Zinc + Riboflavin + Vitamin A (factorial trial) | n=186 total participants analyzed; n=92 treatment, n=94 control | Cognitive |

| Author & Year | Country | Age | Intervention | Comparison group | Sample size | Outcomes |
|---|---|---|---|---|---|---|
| Bora et al, 2019 | India | 0 mo | 2 mg/kg elemental Fe as ferrous ascorbate daily for 6 months | Placebo | n=180 total participants analyzed; n=90 treatment, n=90 control | Hemoglobin, Ferritin |
| Bruner et al, 1996 | USA | 13 yr - 19 yr | 260 mg elemental Fe as ferrous sulfate daily for 8 weeks | Placebo | n=73 total participants analyzed; n=37 treatment, n=36 control | Hemoglobin, Ferritin |
| Burman et al, 1972 | United Kingdom | ~3 mo (mean age) | 10 mg elemental Fe as colloidal ferric hydroxide daily for 21 months | Placebo | n=192 total participants analyzed; n=86 treatment, n=106 control | Hemoglobin |
| Buzina-Suboticanec et al, 1998 | Croatia | 8 yr - 10 yr | 100 mg elemental Fe as ferri-glycine sulfate 6 times per week for 10 weeks | Placebo | n=60 total participants analyzed; n=31 treatment, n=29 control | Hemoglobin, Cognitive |
| Charoenlarp et al, 1980 | Thailand | 6 yr - 13 yr | 40 mg elemental Fe as ferrous sulfate 5 times per week for 5 months | Placebo | n=72 total participants analyzed; n=33 treatment, n=39 control | Hemoglobin, Helminth Infection |
| Chen et al, 2011 | China | 3 yr - 7 yr | 12 mg elemental Fe as NaFeEDTA + Vitamin A 5 times per week for 26 weeks | Placebo + Vitamin A | n=132 total participants analyzed; n=71 treatment, n=61 control | Hemoglobin, Ferritin |
| Chen et al, 2013 | China | 3 yr - 7 yr | 1.5 mg/kg elemental Fe as ferrous sulfate 5 times per week for 6 months | Placebo; or Placebo + Vitamin A (factorial trial) | n=387 total participants analyzed; n=188 treatment, n=199 control | Diarrhea, Respiratory infection |
| Chen et al, 2014 | China | 3 yr - 6 yr | 1-2 mg/kg elemental Fe as ferrous sulfate 5 times per week for 6 months | Control; or Control + Vitamin A (factorial trial) | n=387 total participants analyzed; n=188 treatment, n=199 control | Hemoglobin, Ferritin, Anemia, Iron deficiency |
| Cheng et al, 2001 | China | 7 yr - 12 yr | 2.6 mg elemental Fe as daily for 1 month | Placebo | n=108 total participants analyzed; n=53 treatment, n=55 control | Hemoglobin |

| Author & Year | Country | Age | Intervention | Comparison group | Sample size | Outcomes |
|---|---|---|---|---|---|---|
| Choe et al, 1999 | Korea | 10 yr - 18 yr | 6 mg/kg elemental Fe as ferrous sulfate + H. pylori therapy (bismuth subcitrate, amoxicillin, metronidazole) daily for 10 weeks | Placebo + H. pylori therapy (bismuth subcitrate, amoxicillin, metronidazole) | n=11 total participants analyzed; n=6 treatment, n=5 control | Hemoglobin, Ferritin |
| Chwang et al, 1988 | Indonesia | 8 yr - 14 yr | 2 mg/kg elemental Fe as ferrous sulfate 5 times per week for 12 weeks | Placebo | n=119 total participants analyzed; n=59 treatment, n=60 control | Hemoglobin, HAZ, WAZ |
| Das et al, 1984 | India | 6 mo - 7 yr | 5 mg elemental Fe as neoferrum infants drop 5 times per week for 12 weeks; 10 mg elemental Fe as neoferrum infants drop 5 times per week for 12 weeks; 20 mg elemental Fe as neoferrum infants drop 2 times per week for 12 weeks; 40 mg elemental Fe as neoferrum infants drop 2 times per week for 12 weeks; 20 mg elemental Fe as neoferrum infants drop 1 time per week for 12 weeks; 40 mg elemental Fe as neoferrum infants drop 1 time per week for 12 weeks | Placebo | n=151 total participants analyzed; n=115 treatment, n=36 control | Hemoglobin |
| de Silva et al, 2003 | Sri Lanka | 5 yr - 11 yr | 60 mg elemental Fe as ferrous sulfate daily for 8 weeks | Placebo | n=363 total participants analyzed; n=261 treatment, n=102 control | Hemoglobin, Ferritin, Anemia, Iron deficiency, Diarrhea, Respiratory infection |

| Author & Year | Country | Age | Intervention | Comparison group | Sample size | Outcomes |
|---|---|---|---|---|---|---|
| Desai et al, 2003 | Kenya | 2 mo - 3 yr | 3.83 mg/kg elemental Fe as ferrous sulfate daily for 12 weeks | Placebo; or Placebo + Antimalarial (sulfadoxine-pyrimethamine at 4 and 8 weeks) (factorial trial) | n=491 total participants analyzed; n=256 treatment, n=235 control | Hemoglobin, Anemia, Malaria |
| Devaki et al, 2008 | India | 15 yr - 19 yr | 100 mg elemental Fe as iron(iii) hydroxide polymaltose complex 6 times per week for 8 months | Placebo | n=60 total participants analyzed; n=30 treatment, n=30 control | Hemoglobin, Ferritin |
| Dewey et al, 2002 | Sweden | ~6 mo (mean age) | 1 mg/kg elemental Fe as ferrous sulfate daily for 3 months; 1 mg/kg elemental Fe as ferrous sulfate daily for 5 months; 1 mg/kg elemental Fe as ferrous sulfate daily for 3 months; 1 mg/kg elemental Fe as ferrous sulfate daily for 5 months | Placebo | n=172 total participants analyzed; n=136 treatment, n=36 control | HAZ, WAZ, Diarrhea |
| Dijkhuizen et al, 2001 | Indonesia | ~4 mo (mean age) | 10 mg elemental Fe as ferrous sulfate 5 times per week for 6 months | Placebo; or Placebo + Zinc (factorial trial) | n=360 total participants analyzed; n=172 treatment, n=188 control | Hemoglobin, Ferritin, Anemia, Iron deficiency anemia, HAZ, WHZ, WAZ, Stunting, Wasting |
| Dijkhuizen et al, 2008 | Indonesia, Thailand, Vietnam | 4 mo - 6 mo | 10 mg elemental Fe as ferrous sulfate + Zinc to half the population 5-7 times per week for 6 months | Placebo | n=2451 total participants analyzed; n=1212 treatment, n=1239 control | HAZ, WHZ, WAZ, Stunting, Wasting, Underweight |

| Author & Year | Country | Age | Intervention | Comparison group | Sample size | Outcomes |
|---|---|---|---|---|---|---|
| Domellöf et al, 2001 | Sweden | ~6 mo (mean age) | 1 mg/kg elemental Fe as ferrous sulfate daily for 3 months; 1 mg/kg elemental Fe as ferrous sulfate daily for 5 months; 1 mg/kg elemental Fe as ferrous sulfate daily for 3 months; 1 mg/kg elemental Fe as ferrous sulfate daily for 5 months | Placebo | n=171 total participants analyzed; n=136 treatment, n=35 control | Hemoglobin, Ferritin |
| Dossa et al, 2001 | Benin | 3 yr - 6 yr | 60 mg elemental Fe as ferrous sulfate daily for 3 months | Placebo; or Placebo + Albendazole (factorial trial) | n=140 total participants analyzed; n=70 treatment, n=70 control | Hemoglobin, HAZ, WHZ |
| Dossa et al, 2001 | Benin | 18 mo - 30 mo | 66 mg elemental Fe as ferrous fumarate daily for 6 weeks | Placebo | n=74 total participants analyzed; n=35 treatment, n=39 control | Hemoglobin |
| Eftekhari et al, 2006 | Iran | 14 yr - 19 yr | 60 mg elemental Fe as ferrous sulfate 5 times per week for 12 weeks | Placebo; or Placebo + Iodine (factorial trial) | n=94 total participants analyzed; n=47 treatment, n=47 control | Hemoglobin, Ferritin |
| Elwood et al, 1970 | United Kingdom | 14 yr - 15 yr | 10 mg elemental Fe as ferrous fumarate daily for 36 months; 30 mg elemental Fe as ferrous fumarate daily for 36 months | Placebo + Riboflavin | n=601 total participants analyzed; n=386 treatment, n=215 control | Hemoglobin |
| Engstrom et al, 2008 | Brazil | 5 mo - 7 mo | 12.5 mg elemental Fe as ferrous sulfate daily for 24 weeks; 25 mg elemental Fe as ferrous sulfate 1 time per week for 24 weeks | Placebo | n=391 total participants analyzed; n=297 treatment, n=94 control | Hemoglobin, Anemia |

| Author & Year | Country | Age | Intervention | Comparison group | Sample size | Outcomes |
|---|---|---|---|---|---|---|
| Ermis et al, 2002 | Turkey | ~5 mo (mean age) | 1 mg/kg elemental Fe as ferrous sulfate every other day for 4 months; 2 mg/kg elemental Fe as ferrous sulfate every other day for 4 months; 2 mg/kg elemental Fe as ferrous sulfate every other day for 4 months | Placebo | n=107 total participants analyzed; n=84 treatment, n=23 control | Hemoglobin, Ferritin, Iron deficiency, Iron deficiency anemia |
| Fahmida et al, 2007 | Indonesia | 3 mo - 6 mo | 10 mg elemental Fe as ferrous sulfate + Zinc + Vitamin A daily for 6 months | Placebo + Zinc + Vitamin A | n=377 total participants analyzed; n=186 treatment, n=191 control | Hemoglobin, Ferritin, Anemia, HAZ, WHZ, WAZ, Stunting, Wasting, Underweight |
| Fallahi et al, 2007 | Iran | ~11 yr (mean age) | 20 mg elemental Fe as ferrous sulfate + Zinc 6 times per week for 4 months | Placebo + Zinc | n=54 total participants analyzed; n=26 treatment, n=28 control | Hemoglobin, Ferritin |
| Franz et al, 2000 | Germany | ~0 mo (mean age) | 2-4 mg/kg elemental Fe as ferrous sulfate daily for 8 weeks | Control | n=133 total participants analyzed; n=68 treatment, n=65 control | Ferritin, Iron deficiency |
| Friel et al, 2003 | Canada | ~1 mo (mean age) | 7.5 mg elemental Fe as ferrous sulfate daily for 5 months | Placebo | n=49 total participants analyzed; n=28 treatment, n=21 control | Hemoglobin, Ferritin, Iron deficiency, Iron deficiency anemia |
| Fujiu et al, 2004 | Japan | ~1 mo (mean age) | 4 mg/kg elemental iron (formulation not specified) + Recombinant human erythropoietin daily for 8 weeks | Control + Recombinant human erythropoietin | n=24 total participants analyzed; n=12 treatment, n=12 control | Hemoglobin, Ferritin |
| Gebresellasie, 1996 | Ethiopia | 5 yr - 15 yr | 60 mg elemental Fe as ferrous sulfate daily for 12 weeks | Placebo | n=480 total participants analyzed; n=239 treatment, n=241 control | Hemoglobin, Ferritin, Anemia, Iron deficiency, Malaria |

| Author & Year | Country | Age | Intervention | Comparison group | Sample size | Outcomes |
|---|---|---|---|---|---|---|
| Geltman et al, 2001 | USA | ~6 mo (mean age) | 10 mg elemental iron (formulation not specified) + Multivitamin (A, B, C, D, E) daily for 3 months | Placebo + Multivitamin (A, B, C, D, E) | n=240 total participants analyzed; n=117 treatment, n=123 control | Anemia, Iron deficiency |
| Geltman et al, 2004 | USA | ~6 mo (mean age) | 10 mg elemental Fe as + Multivitamin daily for 3 months | Placebo + Multivitamin | n=284 total participants analyzed; n=138 treatment, n=146 control | Hemoglobin, Ferritin, Anemia, Iron deficiency, Iron deficiency anemia |
| Gokcay et al, 2012 | Turkey | ~6 mo (mean age) | 1 mg/kg elemental Fe as ferrous sulfate + Vitamin D daily for 6 months | Control + Vitamin D | n=105 total participants analyzed; n=51 treatment, n=54 control | Hemoglobin, Anemia |
| Gopaldas et al, 1985 | India | 8 yr - 16 yr | 26.2 mg elemental Fe as ferrous sulfate daily for 2 months; 37.4 mg elemental Fe as ferrous sulfate daily for 2 months | Placebo | n=210 total participants analyzed; n=140 treatment, n=70 control | Hemoglobin, Anemia |
| Gopaldas et al, 1985 | India | 8 yr - 16 yr | 30 mg elemental Fe as ferrous sulfate daily for 2 months; 40 mg elemental Fe as ferrous sulfate daily for 2 months | Placebo | n=48 total participants analyzed; n=32 treatment, n=16 control | Cognitive |
| Greisen et al, 1986 | Zambia | 5 yr - 16 yr | 66 mg elemental Fe as ferrous fumarate 5 times per week for 6 weeks | Placebo | n=430 total participants analyzed; n=218 treatment, n=212 control | Hemoglobin |
| Hacıhamdioglu et al, 2013 | Turkey | ~4 mo (mean age) | 10 mg elemental Fe as ferrous sulfate daily for 2 months | Control | n=53 total participants analyzed; n=27 treatment, n=26 control | Hemoglobin, Ferritin, Iron deficiency |
| Harvey et al, 1989 | Papua New Guinea | 8 yr - 13 yr | 130 mg elemental Fe as ferrous sulfate 5 times per week for 16 weeks | Placebo | n=298 total participants analyzed; n=156 treatment, n=142 control | Hemoglobin, Malaria |



Andersen CT, *et al. BMJ Global Health* 2023; 8:e010745. doi: 10.1136/bmjgh-2022-010745

| Author & Year | Country | Age | Intervention | Comparison group | Sample size | Outcomes |
|---|---|---|---|---|---|---|
| Hathirat et al, 1992 | Thailand | 9 yr - 12 yr | 93.75 mg elemental Fe as ferrous sulfate 5 times per week for 16 weeks | Placebo | n=1772 total participants analyzed; n=885 treatment, n=887 control | Hemoglobin, Ferritin |
| Hess et al, 2002 | Ivory Coast | 5 yr - 15 yr | 60 mg elemental Fe as ferrous sulfate 4 times per week for 16 weeks | Placebo | n=166 total participants analyzed; n=85 treatment, n=81 control | Hemoglobin, Ferritin, Anemia, Iron deficiency |
| Hettiarachchi et al, 2008 | Sri Lanka | 12 yr - 17 yr | 50 mg elemental Fe as ferrous fumarate 5 times per week for 24 weeks | Placebo; or Placebo + Zinc (factorial trial) | n=774 total participants analyzed; n=392 treatment, n=382 control | Hemoglobin, Ferritin, Anemia, Iron deficiency, HAZ, WAZ |
| Hieu et al, 2012 | Vietnam | 6 yr - 10 yr | 1-2 mg/kg elemental Fe as ferrous fumarate 1 time per week for 21-23 weeks | Control | n=221 total participants analyzed; n=95 treatment, n=126 control | Hemoglobin, Ferritin, Anemia, Iron deficiency, Iron deficiency anemia |
| Hop et al, 2005 | Vietnam | 6 mo - 12 mo | 10 mg elemental Fe as ferrous fumarate 7 times per week for 6 months | Placebo | n=152 total participants analyzed; n=75 treatment, n=77 control | Hemoglobin, Ferritin, Anemia, Iron deficiency, HAZ, WHZ, WAZ |
| Htet et al, 2019 | Myanmar | ~16 yr (mean age) | 60 mg elemental iron (formulation not specified) 1 time per week for 12 weeks | Placebo + Folic acid; or Placebo + Vitamin A + Folic acid (factorial trial) | n=391 total participants analyzed; n=192 treatment, n=199 control | Hemoglobin, Ferritin |
| Idjradinata et al, 1993 | Indonesia | 12 mo - 18 mo | 3 mg/kg elemental Fe as ferrous sulfate daily for 4 months | Placebo | n=119 total participants analyzed; n=60 treatment, n=59 control | Hemoglobin, Ferritin |
| Idjratinata et al, 1994 | Indonesia | 12 mo - 18 mo | 3 mg/kg elemental Fe as ferrous sulfate daily for 16 weeks | Placebo | n=44 total participants analyzed; n=22 treatment, n=22 control | HAZ, WAZ |

| Author & Year | Country | Age | Intervention | Comparison group | Sample size | Outcomes |
|---|---|---|---|---|---|---|
| Irigoyen et al, 1991 | USA | ~6 mo (mean age) | 3 mg/kg elemental Fe as ferrous sulfate daily for 3 months; 6 mg/kg elemental Fe as ferrous sulfate daily for 3 months | Placebo | n=225 total participants analyzed; n=150 treatment, n=75 control | Hemoglobin, Ferritin, Diarrhea |
| Jalambo et al, 2018 | Palestine | 15 yr - 20 yr | 200 mg elemental Fe as ferrous fumarate 1 time per week for 3 months | Control | n=87 total participants analyzed; n=45 treatment, n=42 control | Hemoglobin, Ferritin |
| Kapur et al, 2003 | India | 9 mo - 3 yr | 20 mg elemental Fe as ferium, m/s emcure pharm india 1 time per week for 8 weeks | Placebo; or Placebo + Nutrition education (factorial trial) | n=232 total participants analyzed; n=116 treatment, n=116 control | Hemoglobin, Ferritin |
| Kashyap et al, 1987 | India | 8 yr - 15 yr | 60 mg elemental Fe as ferrous sulfate daily for 4 months | Placebo | n=166 total participants analyzed; n=83 treatment, n=83 control | Hemoglobin, Anemia |
| Kashyap et al, 1987 | India | 8 yr - 15 yr | 60 mg elemental Fe as ferrous sulfate daily for 4 months | Placebo | n=130 total participants analyzed; n=65 treatment, n=65 control | Cognitive |
| Kianfar et al, 2000 | Iran | ~16 yr (mean age) | 50 mg elemental Fe as ferrous sulfate daily for 3 months; 50 mg elemental Fe as ferrous sulfate 2 times per week for 3 months; 50 mg elemental Fe as ferrous sulfate 1 time per week for 3 months | Control | n=268 total participants analyzed; n=194 treatment, n=74 control | Hemoglobin, Ferritin |
| Kordas et al, 2005 | Mexico | ~7 yr (mean age) | 30 mg elemental Fe as ferrous fumarate daily for 6 months | Placebo | n=527 total participants analyzed; n=271 treatment, n=256 control | Cognitive |

| Author & Year | Country | Age | Intervention | Comparison group | Sample size | Outcomes |
|---|---|---|---|---|---|---|
| Kusumastuti et al, 2018 | Indonesia | 25 mo - 5 yr | 7.5 mg elemental iron (formulation not specified) daily for 3 months | Placebo; or Placebo + Zinc (factorial trial) | n=68 total participants analyzed; n=34 treatment, n=34 control | HAZ, WAZ |
| Lambert et al, 2002 | New Zealand | 12 yr - 18 yr | 105 mg elemental Fe as ferrous sulfate daily for 8 weeks | Placebo | n=116 total participants analyzed; n=57 treatment, n=59 control | Hemoglobin, Ferritin |
| Latham et al, 1990 | Kenya | ~8 yr (mean age) | 80 mg elemental Fe as ferrous sulfate daily for 15 weeks | Placebo | n=55 total participants analyzed; n=29 treatment, n=26 control | Hemoglobin, Malaria, Helminth Infection |
| Lawless et al, 1994 | Kenya | 6 yr - 12 yr | 47 mg elemental Fe as ferrous sulfate daily for 14 weeks | Placebo | n=86 total participants analyzed; n=44 treatment, n=42 control | Hemoglobin, Ferritin, HAZ, WHZ, WAZ, Diarrhea, Malaria |
| Lee et al, 1997 | Korea | 12 yr - 16 yr | 60 mg elemental Fe as ferrous sulfate daily for 9 weeks | Placebo | n=15 total participants analyzed; n=9 treatment, n=6 control | Hemoglobin, Ferritin |
| Leenstra et al, 2009 | Kenya | 12 yr - 19 yr | 120 mg elemental Fe as ferrous sulfate + Vitamin A 1 time per week for 3 months | Placebo + Vitamin A | n=249 total participants analyzed; n=124 treatment, n=125 control | Hemoglobin, Ferritin, Anemia, Malaria |
| Lind et al, 2003 | Indonesia | ~6 mo (mean age) | 10 mg elemental Fe as ferrous sulfate daily for 6 months | Placebo + Vitamin C; or Placebo + Vitamin C + Zinc (factorial trial) | n=549 total participants analyzed; n=272 treatment, n=277 control | Hemoglobin, Ferritin, Anemia, Iron deficiency, Iron deficiency anemia |
| Lind et al, 2004 | Indonesia | ~6 mo (mean age) | 10 mg elemental Fe as ferrous sulfate daily for 6 months | Placebo + Vitamin C; or Placebo + Zinc + Vitamin C (factorial trial) | n=656 total participants analyzed; n=324 treatment, n=332 control | HAZ, WHZ, WAZ, Stunting, Wasting, Underweight |
| Loría et al, 1979 | Mexico | ~2 mo (mean age) | 1-2 mg/kg elemental Fe as ferrous sulfate daily for 16 months | Placebo; or Placebo + Prenatal iron (factorial trial) | n=92 total participants analyzed; n=48 treatment, n=44 control | Hemoglobin |

| Author & Year | Country | Age | Intervention | Comparison group | Sample size | Outcomes |
|---|---|---|---|---|---|---|
| Lozoff et al, 2016 | China | ~2 mo (mean age) | 1 mg/kg elemental Fe as iron proteinsuccinylate oral solution daily for 7.5 months | Placebo; or Placebo + Maternal iron (factorial trial) | n=1276 total participants analyzed; n=648 treatment, n=628 control | Hemoglobin, Ferritin, Anemia, Iron deficiency, Iron deficiency anemia, HAZ, WHZ, WAZ |
| López de Romaña et al, 2005 | Peru | 6 mo - 12 mo | 10 mg elemental Fe as ferrous fumarate 7 times per week for 6 months | Placebo | n=146 total participants analyzed; n=74 treatment, n=72 control | Hemoglobin, Ferritin, Anemia, Iron deficiency, HAZ, WHZ, WAZ, Diarrhea, Respiratory infection |
| Majumdar et al, 2003 | India | 6 mo - 24 mo | 2 mg/kg elemental iron (formulation not specified) daily for 4 months | Placebo | n=100 total participants analyzed; n=50 treatment, n=50 control | Hemoglobin, Ferritin |
| Malan et al, 2015 | South Africa | 6 yr - 12 yr | 50 mg elemental Fe as ferrous sulfate 4 times per week for 8.5 months | Placebo + Vitamin C; or Placebo + n-3 fatty acids + Vitamin C (factorial trial) | n=296 total participants analyzed; n=146 treatment, n=150 control | Diarrhea, Respiratory infection |
| Massaga et al, 2003 | Tanzania | 3 mo - 4 mo | 7.5 mg elemental Fe as ferric ammonium citrate daily for 6 months | Placebo; or Placebo + Amodaquine (antimalarial) (factorial trial) | n=291 total participants analyzed; n=146 treatment, n=145 control | Anemia, Malaria |
| Mebrahtu et al, 2004 | Tanzania | 4 mo - 6 yr | 10 mg elemental Fe as ferrous sulfate + Placebo & Antihelminthic (Mebendazole) to half daily for 12 months | Placebo + Placebo & Antihelminthic (Mebendazole) to half | n=538 total participants analyzed; n=273 treatment, n=265 control | Malaria |
| Mejía et al, 1988 | Guatemala | 12 mo - 9 yr | 3 mg/kg elemental Fe as ferrous sulfate daily for 2 months | Placebo; or Placebo + Vitamin A (factorial trial) | n=99 total participants analyzed; n=54 treatment, n=45 control | Hemoglobin |

| Author & Year | Country | Age | Intervention | Comparison group | Sample size | Outcomes |
|---|---|---|---|---|---|---|
| Menendez et al, 1997 | Tanzania | ~2 mo (mean age) | 12 mg elemental Fe as ferrous glycine sulphate daily for 16 weeks | Placebo; or Placebo + Malaria prophylaxis (Deltaprim) (factorial trial) | n=832 total participants analyzed; n=417 treatment, n=415 control | Malaria |
| Menendez et al, 2004 | Tanzania | ~2 mo (mean age) | 12 mg elemental Fe as ferrous glycine sulphate daily for 4 months | Placebo; or Placebo + Malaria prophylaxis (Deltaprim) (factorial trial) | n=601 total participants analyzed; n=307 treatment, n=294 control | Ferritin, Iron deficiency |
| Metallinos-Katsaras et al, 2004 | Greece | 3 yr - 5 yr | 15 mg elemental Fe as ferrous fumarate + Multivitamin (Vitamins A, B, C, D, E) 5 times per week for 2 months | Placebo + Multivitamin (Vitamins A, B, C, D, E) | n=48 total participants analyzed; n=31 treatment, n=17 control | Hemoglobin, Ferritin |
| Mitra et al, 1997 | Bangladesh | 2 mo - 4 yr | 15 mg elemental Fe as ferrous gluconate + Multivitamin (A, C, D) daily for 15 months | Placebo + Multivitamin (A, C, D) | n=289 total participants analyzed; n=141 treatment, n=148 control | Diarrhea, Respiratory infection |
| Mozaffari-Koshravi et al, 2010 | Iran | 14 yr - 17 yr | 150 mg elemental Fe as ferrous sulfate 1 time per week for 16 weeks | Control | n=193 total participants analyzed; n=96 treatment, n=97 control | Hemoglobin, Ferritin, Anemia, Iron deficiency, Iron deficiency anemia |
| Mwanri et al, 2000 | Tanzania | 9 yr - 12 yr | 40 mg elemental Fe as ferrous sulfate 3 times per week for 12 weeks | Placebo; or Placebo + Vitamin A (factorial trial) | n=136 total participants analyzed; n=68 treatment, n=68 control | Hemoglobin, Anemia |
| Nagpal et al, 2004 | India | 4 mo - 6 mo | 2 mg/kg elemental Fe as ferric ammonium citrate daily for 8 weeks | Placebo | n=43 total participants analyzed; n=19 treatment, n=24 control | Hemoglobin, Ferritin |
| Nair et al, 2017 | India | 4 mo - 6 mo | 2 mg/kg elemental Fe as colloidal iron daily for 7 months | Control | n=44 total participants analyzed; n=22 treatment, n=22 control | Hemoglobin, Anemia |



Andersen CT, *et al. BMJ Global Health* 2023; 8:e010745. doi: 10.1136/bmjgh-2022-010745

| Author & Year | Country | Age | Intervention | Comparison group | Sample size | Outcomes |
|---|---|---|---|---|---|---|
| Nchito et al, 2009 | Zambia | ~10 yr (mean age) | 60 mg elemental Fe as ferrous dextran 5 times per week for 10 months | Placebo; or Placebo + Multivitamin (factorial trial) | n=215 total participants analyzed; n=118 treatment, n=97 control | Helminth Infection |
| Northrop-Clewes et al, 1996 | Pakistan | ~14 mo (mean age) | 15 mg elemental Fe as ferrous sulfate daily for 12 weeks | Placebo | n=191 total participants analyzed; n=95 treatment, n=96 control | Hemoglobin, Ferritin |
| Olsen et al, 2000 | Kenya | 4 yr - 16 yr | 60 mg elemental Fe as ferrous dextran 2 times per week for 12 months | Placebo | n=200 total participants analyzed; n=108 treatment, n=92 control | Helminth Infection |
| Olsen et al, 2006 | Kenya | ~9 yr (mean age) | 60 mg elemental Fe as ferrous dextran 2 times per week for 12 months | Placebo | n=200 total participants analyzed; n=108 treatment, n=92 control | Hemoglobin, Ferritin |
| Paganini et al, 2017 | Kenya | 6 mo - 9.5 mo | 5 mg elemental Fe as NaFeEDTA and ferrous fumarate + Micronutrient powder daily for 4 months | Placebo + Micronutrient powder | n=97 total participants analyzed; n=49 treatment, n=48 control | Hemoglobin, Ferritin, Anemia, Iron deficiency anemia |
| Palupi et al, 1997 | Indonesia | 24 mo - 6 yr | 30 mg elemental Fe as ferrous sulfate 1 time per week for 9 weeks | Placebo | n=194 total participants analyzed; n=96 treatment, n=98 control | Hemoglobin, Anemia, HAZ, WHZ, WAZ, Helminth Infection |
| Paracha et al, 1993 | Pakistan | 8 yr - 11 yr | 76 mg elemental Fe as ferrous gluconate + Multivitamin daily for 11 weeks | Control + Multivitamin | n=119 total participants analyzed; n=61 treatment, n=58 control | Hemoglobin, Ferritin |
| Perrone et al, 1999 | Italy | 4 yr - 12 yr | 12 mg elemental Fe as iron polymaltosate + Zinc daily for 12 months | Control + Zinc | n=20 total participants analyzed; n=10 treatment, n=10 control | Ferritin, HAZ, WAZ |
| Prasetyani et al, 2017 | Indonesia | 5 yr - 19 yr | 100 mg elemental Fe as iron(iii)-hydroxide polymaltose complex 6 times per week for 8 weeks | Placebo | n=578 total participants analyzed; n=290 treatment, n=288 control | Hemoglobin, Ferritin, Malaria |

| Author & Year | Country | Age | Intervention | Comparison group | Sample size | Outcomes |
|---|---|---|---|---|---|---|
| Reeves et al, 1985 | USA | 11 mo - 14 mo | 3 mg/kg elemental Fe as ferrous sulfate daily for 3 months | Placebo | n=179 total participants analyzed; n=77 treatment, n=102 control | Diarrhea |
| Rezaeian et al, 2014 | Iran | 14 yr - 19 yr | 50 mg elemental Fe as ferrous sulfate 2 times per week for 16 weeks | Placebo | n=200 total participants analyzed; n=100 treatment, n=100 control | Hemoglobin |
| Richard et al, 2006 | Peru | 6 mo - 16 yr | 15 mg elemental Fe as ferrous sulfate daily for 7 months | Placebo; or Placebo + Zinc (factorial trial) | n=758 total participants analyzed; n=378 treatment, n=380 control | Hemoglobin, HAZ, WAZ, Diarrhea, Respiratory infection, Malaria |
| Rosado et al, 1997 | Mexico | 18 mo - 3 yr | 20 mg elemental Fe as ferrous sulfate 5 times per week for 12 months | Placebo; or Placebo + Zinc (factorial trial) | n=217 total participants analyzed; n=108 treatment, n=109 control | Hemoglobin, Ferritin, Iron deficiency, HAZ, WHZ, WAZ, Diarrhea, Respiratory infection |
| Rosado et al, 2006 | Mexico | 6 yr - 8 yr | 30 mg elemental Fe as ferrous fumarate 5 times per week for 6 months | Placebo; or Placebo + Zinc (factorial trial) | n=517 total participants analyzed; n=265 treatment, n=252 control | Hemoglobin, Ferritin |
| Roschnik et al, 2004 | Philippines | 7 yr - 13 yr | 108 mg elemental Fe as ferrous sulfate 1 time per week for 10 weeks | Control | n=1510 total participants analyzed; n=708 treatment, n=802 control | Hemoglobin, Anemia |
| Sarker et al, 2008 | Bangladesh | 24 mo - 6 yr | 3 mg/kg elemental Fe as ferrous sulfate daily for 3 months | Placebo; or Placebo + H-pylori treatment (factorial trial) | n=197 total participants analyzed; n=99 treatment, n=98 control | Hemoglobin, Ferritin |
| Seshadri et al, 1989 | India | 8 yr - 16 yr | 30 mg elemental Fe as ferrous sulfate daily for 2 months; 40 mg elemental Fe as ferrous sulfate daily for 2 months | Placebo | n=113 total participants analyzed; n=97 treatment, n=16 control | Hemoglobin, Anemia |
| Smith et al, 1989 | Gambia | 6 mo - 6 yr | 3-6 mg/kg elemental Fe as ferrous sulfate daily for 12 weeks | Placebo | n=186 total participants analyzed; n=97 treatment, n=89 control | Malaria |

| Author & Year | Country | Age | Intervention | Comparison group | Sample size | Outcomes |
|---|---|---|---|---|---|---|
| Smuts et al, 2005 | South Africa | 6 mo - 12 mo | 10 mg elemental iron (formulation not specified) daily for 6 months | Placebo | n=99 total participants analyzed; n=49 treatment, n=50 control | Hemoglobin, Ferritin, HAZ, WHZ, WAZ, Diarrhea, Respiratory infection |
| Smuts et al, 2014 | South Africa | 6 yr - 12 yr | 50 mg elemental Fe as ferrous sulfate 4 times per week for 8.5 months | Placebo + Vitamin C; or Placebo + n-3 fatty acids + Vitamin C (factorial trial) | n=86 total participants analyzed; n=43 treatment, n=43 control | Cognitive |
| Soemantri et al, 1989 | Indonesia | 8 yr - 12 yr | 2 mg/kg elemental Fe as ferrous sulfate daily for 3 months | Placebo | n=130 total participants analyzed; n=71 treatment, n=59 control | Hemoglobin, Cognitive |
| Soewondo et al, 1989 | Indonesia | ~5 yr (mean age) | 50 mg elemental Fe as ferrous sulfate daily for 8 weeks | Placebo | n=176 total participants analyzed; n=77 treatment, n=99 control | Hemoglobin, Ferritin |
| Stoltzfus et al, 2001 | Tanzania (Zanzibar) | 12 mo - 4 yr | 10 mg elemental Fe as ferrous sulfate daily for 12 months | Placebo | n=538 total participants analyzed; n=277 treatment, n=261 control | Hemoglobin, Ferritin, Helminth Infection, Cognitive |
| Stoltzfus et al, 2004 | Tanzania | 6 mo - 29 mo | 10 mg elemental Fe as ferrous sulfate daily for 12 months | Placebo; or Placebo + Mebendezole (factorial trial) | n=145 total participants analyzed; n=74 treatment, n=71 control | Anemia, Stunting |
| Sungthong et al, 2002 | Thailand | 6 yr - 14 yr | 60 mg elemental Fe as ferrous sulfate daily for 16 weeks; 60 mg elemental Fe as ferrous sulfate 1 time per week for 16 weeks | Placebo | n=396 total participants analyzed; n=274 treatment, n=122 control | Hemoglobin, Ferritin, Iron deficiency anemia, HAZ, WAZ |

| Author & Year | Country | Age | Intervention | Comparison group | Sample size | Outcomes |
|---|---|---|---|---|---|---|
| Sungthong et al, 2004 | Thailand | 6 yr - 14 yr | 60 mg elemental Fe as ferrous sulfate 5 times per week for 16 weeks; 60 mg elemental Fe as ferrous sulfate 1 time per week for 16 weeks | Placebo | n=391 total participants analyzed; n=269 treatment, n=122 control | Cognitive |
| Teshome et al, 2017 | Kenya | 12 mo - 3 yr | 3 mg elemental Fe as NaFeEDTA daily for 1 month; 12.5 mg elemental Fe as ferrous fumarate daily for 1 month | Placebo + Anti-malarial (dihydroartemisinin-piperaquine); anti-helminthic (albendazole, praziquantel); micronutrient powder (13 nutrients) | n=315 total participants analyzed; n=210 treatment, n=105 control | Hemoglobin, Ferritin, Anemia, Iron deficiency, Malaria |
| Thibault et al, 1993 | France | 6 mo - 4 yr | 38.6 mg elemental Fe as hydroxyproline iron daily for 2 months | Placebo | n=70 total participants analyzed; n=32 treatment, n=38 control | Hemoglobin, Ferritin |
| Untoro et al, 2005 | Indonesia | 6 mo - 12 mo | 10 mg elemental Fe as ferrous sulfate 7 times per week for 23 weeks | Placebo | n=134 total participants analyzed; n=69 treatment, n=65 control | Hemoglobin, Ferritin, Anemia, Iron deficiency, HAZ, WHZ, WAZ, Diarrhea, Respiratory infection |
| van den Hombergh et al, 1996 | Tanzania | 0 mo - 30 mo | 40 mg elemental Fe as ferrous sulfate + Malaria treatment + folic acid daily for 12 weeks | Placebo + Malaria treatment + folic acid | n=95 total participants analyzed; n=48 treatment, n=47 control | Hemoglobin, WAZ, Malaria |
| Verhoef et al, 2002 | Kenya | 2 mo - 3 yr | 3 mg/kg elemental Fe as ferrous fumarate 2 times per week for 12 weeks | Placebo; or Placebo + Antimalarial (sulfadoxine- | n=328 total participants analyzed; n=164 treatment, n=164 control | Hemoglobin, Iron deficiency, Malaria |

| **Author & Year** | **Country** | **Age** | **Intervention** | **Comparison group** | **Sample size** | **Outcomes** |
|---|---|---|---|---|---|---|
| | | | | pyrimethamine) (factorial trial) | | |
| Wang et al, 2012 | China | ~4 mo (mean age) | 1 mg/kg elemental Fe as  for 2 months | Control | n=60 total participants analyzed; n=26 treatment, n=34 control | Hemoglobin, Ferritin |
| Wasantwisut et al, 2006 | Thailand | 4 mo - 6 mo | 10 mg elemental Fe as ferrous sulfate daily for 6 months | Placebo + Vitamin C, A; or Placebo + Zinc + Vitamins C, A (factorial trial) | n=609 total participants analyzed; n=305 treatment, n=304 control | Hemoglobin, Ferritin, Anemia, Iron deficiency, Iron deficiency anemia, HAZ, WHZ, WAZ |
| Wieringa et al, 2007 | Indonesia, Thailand, Vietnam | 4 mo - 6 mo | 10 mg elemental Fe as ferrous sulfate 5-7 times per week for 6 months | Placebo; or Placebo + Zinc (factorial trial) | n=2049 total participants analyzed; n=1017 treatment, n=1032 control | Hemoglobin, Ferritin, Anemia, Iron deficiency, Iron deficiency anemia |
| Yalçin et al, 2000 | Turkey | ~6 mo (mean age) | 1 mg/kg elemental Fe as ferrous sulfate daily for 3 months | Control | n=16 total participants analyzed; n=7 treatment, n=9 control | Hemoglobin, Ferritin, Anemia, Iron deficiency, Iron deficiency anemia |
| Yip et al, 1985 | USA | 11 mo - 13 mo | 30 mg/kg elemental Fe as ferrous sulfate daily for 3 months | Placebo | n=291 total participants analyzed; n=146 treatment, n=145 control | Hemoglobin, Ferritin |
| Yurdakök et al, 2004 | Turkey | ~4 mo (mean age) | 1 mg/kg elemental Fe as ferrous sulfate daily for 3 months; 7 mg/kg elemental Fe as ferrous sulfate 1 time per week for 3 months | Control | n=53 total participants analyzed; n=37 treatment, n=16 control | Hemoglobin, Ferritin |
| Zavaleta et al, 2000 | Peru | 12 yr - 19 yr | 60 mg elemental Fe as ferrous sulfate 2 times per week for 17 weeks | Placebo | n=198 total participants analyzed; n=101 treatment, n=97 control | Anemia |

| **Author & Year** | **Country** | **Age** | **Intervention** | **Comparison group** | **Sample size** | **Outcomes** |
|---|---|---|---|---|---|---|
| Zhao et al, 2004 | China | 3 yr - 6 yr | 4 mg elemental Fe as NaFeEDTA for 18 months | Placebo | n=213 total participants analyzed; n=120 treatment, n=93 control | Hemoglobin, Anemia |
| Ziegler et al, 2009 | USA | 4 mo - 4 mo | 7.5 mg elemental Fe as ferrous sulfate daily for 5 months | Control | n=98 total participants analyzed; n=42 treatment, n=56 control | Hemoglobin, Ferritin |
| Ziegler et al, 2009 | USA | ~1 mo (mean age) | 7 mg elemental Fe as ferrous sulfate + Multivitamins (A, C, D) daily for 4.5 months | Placebo + Multivitamins (A, C, D) | n=63 total participants analyzed; n=31 treatment, n=32 control | Hemoglobin, Ferritin, Iron deficiency, Iron deficiency anemia |
| Zlotkin et al, 2003 | Ghana | 8 mo - 20 mo | 40 mg elemental Fe as ferrous fumarate daily for 6 months; 12.5 mg elemental Fe as ferrous sulfate daily for 6 months | Placebo | n=241 total participants analyzed; n=161 treatment, n=80 control | Hemoglobin, Ferritin, Anemia |
| Zlotkin et al, 2013 | Ghana | 6 mo - 4 yr | 12.5 mg elemental Fe as microencapsulated ferrous fumerate + Ascorbic acid + vitamin a + zinc daily for 5 months | Placebo + Ascorbic acid + vitamin a + zinc | n=1815 total participants analyzed; n=900 treatment, n=915 control | Hemoglobin, Anemia, Iron deficiency, Malaria |



Andersen CT, *et al. BMJ Global Health* 2023; 8:e010745. doi: 10.1136/bmjgh-2022-010745

**Appendix 6.** Modification of iron effects after control for baseline anemia.

| | Hemoglobin (g/L) * | Ferritin (ng/mL) * | Anemia † | Iron deficiency † | Iron deficiency anemia † |
|---|---|---|---|---|---|
| **Weekly frequency** | | | | | |
| Frequent (3-7 times/week) | ref | ref | ref | ref | ref |
| n | 95 | 60 | 48 | 29 | 15 |
| Intermittent (1-2 times/week) | | | | | |
| n | 23 | 16 | 9 | 4 | 3 |
| Estimate | -3.1 | -15.0 | 0.98 | 1.12 | 1.61 |
| 95% CI | (-5.9, -0.3) | (-24.7, -5.3) | (0.65, 1.48) | (0.24, 3.19) | (0.29, 8.84) |
| p-interaction | 0.03 | 0.003 | 0.94 | 0.83 | 0.56 |
| **Duration** | | | | | |
| 1-3 months | | | | | |
| n | 74 | 43 | 23 | 13 | 5 |
| Estimate | ref | ref | ref | ref | ref |
| 4-6 months | | | | | |
| n | 39 | 30 | 30 | 20 | 13 |
| Estimate | 0.2 | 8.2 | 1.07 | 0.55 | 1.54 |
| 95% CI | (-2.4, 2.9) | (-1.0, 17.5) | (0.76, 1.52) | (0.25, 1.25) | (0.26, 9.28) |
| ≥7 months | | | | | |
| n | 8 | 4 | 6 | 0 | 0 |
| Estimate | -1.3 | 40.7 | 1.38 | n/e | n/e |
| 95% CI | (-6.0, 3.5) | (1.7, 79.6) | (0.85, 2.24) | | |
| Global p-interaction | 0.83 | 0.04 | 0.40 | 0.15 | 0.61 |
| **Dose‡** | | | | | |
| Low (1st tertile) | | | | | |
| n | 36 | 26 | 21 | 9 | 8 |
| Estimate | ref | ref | ref | ref | ref |
| Moderate (2nd tertile) | | | | | |
| n | 27 | 21 | 22 | 15 | 9 |
| Estimate | 4.9 | 14.5 | 0.82 | 1.01 | 0.30 |
| 95% CI | (1.8, 8.0) | (5.9, 23.1) | (0.59, 1.13) | (0.40, 2.57) | (0.14, 0.69) |
| High (3rd tertile) | | | | | |
| n | 31 | 20 | 8 | 6 | 1 |
| Estimate | 2.1 | 6.4 | 1.07 | 0.96 | 0.24 |
| 95% CI | (-1.0, 5.1) | (-2.4, 15.1) | (0.68, 1.70) | (0.24, 3.93) | (0.01, 6.80) |
| Global p-interaction | 0.008 | 0.006 | 0.33 | 1.00 | 0.008 |

* Difference in the weighted mean effect of iron in the index category compared to the weighted mean effect of iron in the reference category, adjusting for baseline anemia.

† Ratio of the prevalence ratio for iron in the index category relative to the prevalence ratio for iron in the reference category, adjusting for baseline anemia.

‡ Age-adjusted dose tertiles; see Appendix 3.

Abbreviations: CI, confidence interval; ref, reference category; n/e, not estimable

**Appendix 7.** Effect modification of iron by baseline anemia, child age, child sex, WHO region, and iron formulation.

| | Hemoglobin (g/L) * | Ferritin (ng/mL) * | Anemia † | Iron deficiency † | Iron deficiency anemia † |
|---|---|---|---|---|---|
| **Baseline anemia** | | | | | |
| All anemic | | | | | |
| n | 33 | 17 | 9 | 5 | 1 |
| Estimate | 10.6 | 13.1 | 0.35 | 0.19 | 0.15 |
| 95% CI | (7.3, 13.9) | (8.0, 18.2) | (0.26, 0.47) | (0.06, 0.61) | (0.02, 1.26) |
| Mixed anemic and non-anemic | | | | | |
| n | 54 | 30 | 40 | 23 | 13 |
| Estimate | 6.5 | 22.7 | 0.63 | 0.26 | 0.13 |
| 95% CI | (5.4, 7.5) | (17.4, 27.9) | (0.55, 0.71) | (0.19, 0.36) | (0.08, 0.22) |
| All non-anemic | | | | | |
| n | 28 | 20 | 10 | 5 | 4 |
| Estimate | 5.1 | 18.7 | 0.62 | 0.20 | 0.21 |
| 95% CI | (3.5, 6.7) | (13.5, 23.9) | (0.41, 0.93) | (0.11, 0.36) | (0.06, 0.70) |
| Missing baseline anemia data | | | | | |
| n | 52 | 40 | 10 | 15 | 9 |
| Estimate | 3.3 | 16.6 | 0.82 | 0.61 | 0.48 |
| 95% CI | (2.4, 4.2) | (13.4, 19.8) | (0.70, 0.95) | (0.48, 0.78) | (0.29, 0.78) |
| Global p-interaction | <0.001 | 0.28 | 0.002 | 0.13 | 0.06 |
| **Child age** | | | | | |
| 0 to 5 months | | | | | |
| n | 39 | 33 | 18 | 19 | 17 |
| Estimate | 5.2 | 25.1 | 0.57 | 0.23 | 0.17 |
| 95% CI | (3.7, 6.7) | (19.7, 30.5) | (0.48, 0.70) | (0.15, 0.35) | (0.10, 0.31) |
| 6 to 23 months | | | | | |
| n | 31 | 23 | 23 | 15 | 5 |
| Estimate | 5.1 | 14.4 | 0.77 | 0.38 | 0.32 |
| 95% CI | (3.3, 6.9) | (8.7, 20.0) | (0.67, 0.90) | (0.27, 0.53) | (0.14, 0.69) |
| 2 to <5 years | | | | | |
| n | 25 | 10 | 6 | 4 | 0 |
| Estimate | 5.7 | 19.5 | 0.76 | 0.31 | n/e |
| 95% CI | (3.8, 7.7) | (11.3, 27.7) | (0.53, 1.09) | (0.14, 0.67) | |
| 5 to <12 years | | | | | |
| n | 29 | 13 | 13 | 6 | 4 |
| Estimate | 8.5 | 17.9 | 0.48 | 0.27 | 0.19 |
| 95% CI | (5.7, 11.4) | (10.9, 24.9) | (0.38, 0.60) | (0.11, 0.69) | (0.06, 0.56) |
| ≥12 years | | | | | |
| n | 21 | 20 | 5 | 3 | 1 |
| Estimate | 6.6 | 10.9 | 0.49 | 0.30 | 0.09 |
| 95% CI | (4.4, 8.7) | (9.1, 12.7) | (0.31, 0.79) | (0.11, 0.80) | (0.01, 1.64) |
| Global p-interaction | 0.18 | 0.01 | 0.047 | 0.73 | 0.57 |
| **Child sex** | | | | | |

| | | | | | |
|---|---|---|---|---|---|
| All female | | | | | |
| n | 22 | 17 | 5 | 2 | 1 |
| Estimate | 6.6 | 9.3 | 0.39 | 0.74 | 0.09 |
| 95% CI | (4.2, 9.1) | (7.7, 10.9) | (0.28, 0.55) | (0.46, 1.19) | (0.01, 1.64) |
| All male | | | | | |
| n | 6 | 0 | 2 | 0 | 0 |
| Estimate | 12.1 | n/e | 0.54 | n/e | n/e |
| 95% CI | (10.1, 14.2) | | (0.46, 0.64) | | |
| Mixed female and male | | | | | |
| n | 100 | 70 | 58 | 38 | 25 |
| Estimate | 5.5 | 20.1 | 0.63 | 0.33 | 0.21 |
| 95% CI | (4.6, 6.4) | (16.8, 23.5) | (0.57, 0.70) | (0.26, 0.41) | (0.13, 0.32) |
| Missing baseline sex data | | | | | |
| n | 39 | 20 | 4 | 8 | 1 |
| Estimate | 7.6 | 21.5 | 0.61 | 0.17 | 0.11 |
| 95% CI | (4.9, 10.3) | (12.3, 30.6) | (0.43, 0.86) | (0.09, 0.31) | (0.01, 1.99) |
| Global p-interaction | 0.07 | 0.04 | 0.41 | 0.14 | 0.85 |
| **WHO region** | | | | | |
| Africa | | | | | |
| n | 26 | 11 | 20 | 10 | 2 |
| Estimate | 6.1 | 17.6 | 0.70 | 0.28 | 0.35 |
| 95% CI | (3.9, 8.3) | (13.1, 22.1) | (0.60, 0.82) | (0.16, 0.49) | (0.21, 0.56) |
| Americas | | | | | |
| n | 29 | 13 | 13 | 7 | 3 |
| Estimate | 4.6 | 19.3 | 0.63 | 0.75 | 0.66 |
| 95% CI | (2.6, 6.7) | (13.1, 25.5) | (0.43, 0.91) | (0.51, 1.09) | (0.20, 2.14) |
| Eastern Mediterranean | | | | | |
| n | 12 | 11 | 1 | 1 | 1 |
| Estimate | 8.6 | 8.9 | 0.14 | 0.79 | 0.09 |
| 95% CI | (6.1, 11.2) | (5.8, 12.0) | (0.03, 0.62) | (0.48, 1.29) | (0.01, 1.64) |
| Europe | | | | | |
| n | 21 | 19 | 5 | 10 | 6 |
| Estimate | 4.4 | 17.4 | 0.32 | 0.28 | 0.16 |
| 95% CI | (2.8, 6.0) | (11.8, 23.1) | (0.14, 0.70) | (0.20, 0.41) | (0.06, 0.45) |
| South-East Asia | | | | | |
| n | 56 | 34 | 20 | 12 | 10 |
| Estimate | 8.2 | 25.4 | 0.57 | 0.23 | 0.13 |
| 95% CI | (6.5, 9.7) | (19.4, 31.5) | (0.48, 0.67) | (0.16, 0.33) | (0.09, 0.18) |
| Western Pacific | | | | | |
| n | 18 | 14 | 10 | 8 | 5 |
| Estimate | 5.4 | 14.1 | 0.58 | 0.38 | 0.53 |
| 95% CI | (3.6, 7.3) | (8.3, 19.9) | (0.44, 0.76) | (0.25, 0.57) | (0.29, 0.96) |
| Global p-interaction | 0.06 | 0.06 | 0.20 | 0.44 | 0.007 |
| **Iron formulation** | | | | | |
| Ferrous sulfate | | | | | |
| n | 110 | 66 | 46 | 27 | 20 |
| Estimate | 7.2 | 21.8 | 0.56 | 0.21 | 0.12 |

| | | | | | |
|---|---|---|---|---|---|
| 95% CI | (6.1, 8.3) | (18.5, 25.1) | (0.49, 0.64) | (0.15, 0.30) | (0.09, 0.17) |
| Ferrous fumarate | | | | | |
| n | 15 | 11 | 8 | 9 | 1 |
| Estimate | 4.8 | 16.7 | 0.69 | 0.29 | 0.40 |
| 95% CI | (2.2, 7.4) | (12.7, 20.8) | (0.53, 0.91) | (0.18, 0.47) | (0.08, 2.01) |
| Other or unspecified | | | | | |
| n | 41 | 29 | 14 | 12 | 5 |
| Estimate | 4.2 | 10.3 | 0.78 | 0.56 | 0.81 |
| 95% CI | (2.8, 5.6) | (5.3, 15.3) | (0.62, 0.97) | (0.44, 0.73) | (0.58, 1.14) |
| Global p-interaction | 0.02 | 0.005 | 0.13 | 0.03 | <0.001 |

* Weighted mean difference
† Pooled risk ratio
Abbreviations: CI, confidence interval; n/e, not estimable

**Appendix 8.** Comparison of iron supplementation effects within factorial trials, using double placebo as a common referent group.

| | Hemoglobin (g/L) * | Ferritin (ng/mL) * | Anemia † | Iron deficiency † | Iron deficiency anemia † |
|---|---|---|---|---|---|
| **Zinc** | | | | | |
| n | 10 | 9 | 6 | 6 | 5 |
| Double placebo | ref | ref | ref | ref | ref |
| Iron + Placebo | 6.6 (3.6, 9.6) | 28.8 (22.2, 35.4) | 0.41 (0.33, 0.50) | 0.15 (0.09, 0.24) | 0.08 (0.05, 0.15) |
| Zinc + Placebo | 0.1 (-1.5, 1.6) | -0.4 (-2.0, 1.2) | 0.93 (0.78, 1.10) | 1.06 (0.95, 1.17) | 1.04 (0.81, 1.34) |
| Iron + Zinc | 4.2 (1.9, 6.5) | 21.0 (16.7, 25.3) | 0.59 (0.46, 0.75) | 0.19 (0.12, 0.31) | 0.16 (0.09, 0.27) |
| **Vitamin A** | | | | | |
| n | 6 | 3 | 2 | 1 | 0 |
| Double placebo | ref | ref | ref | ref | n/a |
| Iron + Placebo | 7.6 (3.0, 12.2) | 8.4 (5.3, 11.4) | 0.27 (0.16, 0.45) | 0.34 (0.18, 0.66) | n/a |
| Vitamin A + Placebo | 6.0 (3.2, 8.8) | -4.0 (-17.6, 9.5) | 0.49 (0.36, 0.67) | 1.24 (0.83, 1.84) | n/a |
| Iron + Vitamin A | 10.5 (6.0, 14.9) | 0.6 (-4.1, 5.3) | 0.25 (0.07, 0.94) | 0.63 (0.38, 1.07) | n/a |

* Weighted mean difference

† Pooled risk ratio

Abbreviations: CI, confidence interval

**Appendix 9.** Effect heterogeneity p-values for anthropometric, infectious, and development outcomes.*†

| | Modifiers | | | | | | | |
|---|---|---|---|---|---|---|---|---|
| | Frequency | Duration | Dose for age tertile | Baseline percent anemic | Age category | Baseline percent female | Factorial trial | Formulation |
| *Anthropometry* | | | | | | | | |
| Height-for-age Z score | 0.255 | 0.598 | 0.835 | 0.013 | 0.256 | n/e | 0.048 | 0.272 |
| Weight-for-height Z score | 0.970 | 0.665 | 0.291 | n/e | 0.311 | n/e | 0.559 | 0.843 |
| Weight-for-age Z score | 0.524 | 0.508 | 0.861 | 0.709 | 0.250 | n/e | 0.363 | 0.847 |
| Stunting | n/e | 0.452 | 0.393 | n/e | 0.378 | 0.378 | 0.700 | n/e |
| Wasting | n/e | n/e | 0.522 | n/e | n/e | n/e | 0.336 | n/e |
| *Infections* | | | | | | | | |
| Diarrhea (cumulative incidence) | 0.136 | 0.282 | 0.274 | 0.358 | 0.884 | n/e | 0.642 | 0.904 |
| Diarrhea (incidence rate) | 0.962 | 0.471 | 0.435 | n/e | 0.446 | n/e | 0.760 | 0.760 |
| Respiratory illness (cumulative incidence) | 0.006 | 0.018 | 0.032 | 0.125 | n/e | n/e | 0.129 | 0.683 |
| Respiratory illness (incidence rate) | 0.902 | 0.866 | 0.750 | n/e | 0.887 | n/e | 0.421 | 0.421 |
| Malaria (prevalence) | n/e | n/e | 0.996 | 0.910 | n/e | n/e | 0.225 | 0.622 |
| Malaria (incidence rate) | n/e | 0.469 | 0.965 | 0.871 | n/e | n/e | 0.463 | 0.467 |
| Hookworm (prevalence) | 0.968 | 0.812 | 0.880 | n/e | n/e | n/e | n/e | 0.823 |
| Ascaris lumbricoides (prevalence) | 0.390 | 0.456 | 0.647 | n/e | n/e | n/e | n/e | 0.932 |
| Trichuris trichiura (prevalence) | 0.501 | 0.501 | 0.431 | n/e | n/e | n/e | n/e | 0.584 |
| *Development* | | | | | | | | |
| Bayley Mental Index | 0.651 | 0.892 | 0.888 | 0.020 | n/e | n/e | 0.280 | 0.454 |
| Bayley Psychomotor Index | 0.807 | 0.432 | 0.738 | 0.023 | n/e | n/e | 0.672 | 0.634 |

* Categories used for modifiers: Frequency (1-2 times per week vs. 3-7 times per week); Dose for age tertile (1st vs. 2nd vs. 3rd); Baseline percent anemic (<20% anemic vs. 20-39% anemic vs. ≥40% anemic); Age category (0-5 mo vs. 6-23 mo vs. 24-59 mo vs. 5-11 y vs. 12-19 y); Baseline percent female (All participants female vs. no participants female vs. some participants female); Factorial trial (Yes vs. no); Formulation (Ferrous sulfate vs. ferrous fumarate vs. other)

† P-values calculated from meta-regression Wald tests.
Abbreviations: n/e, not estimable.

**Appendix 10.** Risk of bias within included studies.

| Study | Random sequence generation (selection bias) | Allocation concealment (selection bias) | Blinding of partici-pants and personnel (performance bias) | Blinding of outcome assessment (detection bias) | Incomplete outcome data (attrition bias) |
|---|---|---|---|---|---|
| Adish 1997 | Unclear | Unclear | Low | Low | Low |
| Aggarwal 2005 | Low | Unclear | Low | Low | High |
| Aguayo 2000 | Low | Unclear | Low | Low | Low |
| Akman 2004 | Low | Unclear | High | Low | Low |
| Angeles 1993 | Unclear | Unclear | Unclear | Unclear | Low |
| Angulo-Barro 2016 | Low | Low | Low | Low | Unclear |
| Arcanjo 2011 | High | Unclear | Low | Low | Unclear |
| Arcanjo 2013 | High | Unclear | Low | Low | Unclear |
| Aukett 1986 | Unclear | Low | Low | Low | Low |
| Ayoya 2009 | Unclear | Unclear | High | Low | High |
| Ayoya 2012 | Unclear | Unclear | High | Low | High |
| Ballin 1992 | High | High | Low | Low | Unclear |
| Baqui 2003 | Unclear | Unclear | Unclear | Unclear | Low |
| Barclay 1991 | Unclear | Unclear | High | High | Unclear |
| Baumgartner 2012 | Low | Unclear | Low | Low | High |
| Berger 1997 | Unclear | Unclear | Low | Unclear | Low |
| Berger 2000 | Unclear | Unclear | Low | Low | Unclear |
| Berger 2006 | Low | Low | Low | Low | High |
| Berglund 2010 | Low | Unclear | Low | Low | High |
| Bhatia 1993 | High | Unclear | Unclear | Unclear | Unclear |
| Black 2004 | Unclear | Unclear | Low | Low | Low |
| Bora 2019 | Low | Low | Unclear | Unclear | Low |
| Bruner 1996 | Low | Unclear | Low | Low | High |
| Burman 1972 | High | Unclear | High | High | High |
| Buzina-Suboticanec 1998 | High | Unclear | Low | Low | Unclear |
| Charoenlarp 1980 | Unclear | Unclear | Unclear | Unclear | Unclear |
| Chen 2011 | Unclear | High | Unclear | Low | Unclear |
| Chen 2013 | Low | Unclear | Low | Low | Low |
| Chen 2014 | Low | Unclear | High | High | Low |
| Cheng 2001 | Unclear | Unclear | Unclear | Unclear | Unclear |
| Choe 1999 | Low | Unclear | Low | Low | Unclear |
| Chwang 1988 | Unclear | Unclear | Low | Low | Low |
| Das 1984 | High | Unclear | Unclear | Unclear | Low |
| de Silva 2003 | Unclear | Unclear | Low | Low | Unclear |
| Desai 2003 | Low | Low | Low | Low | High |
| Devaki 2008 | Unclear | Unclear | Unclear | Unclear | Low |
| Dewey 2002 | Unclear | Unclear | Low | Low | Low |
| Dijkhuizen 2001 | Low | Low | Low | Low | Low |
| Dijkhuizen 2008 | Low | Low | Low | Low | Unclear |
| Domellöf 2001 | Unclear | Unclear | Low | Low | Unclear |
| Dossa 2001 | Low | Unclear | Low | Low | Low |
| Dossa 2001 | Unclear | Unclear | Low | Low | Unclear |
| Eftekhari 2006 | Unclear | Unclear | Low | Low | High |
| Elwood 1970 | Unclear | Unclear | Unclear | Unclear | Unclear |
| Engstrom 2008 | Unclear | Unclear | High | High | Unclear |
| Ermis 2002 | Unclear | Unclear | Unclear | Unclear | Unclear |
| Fahmida 2007 | Unclear | Unclear | Low | Low | Low |

| | | | | | |
|---|---|---|---|---|---|
| Fallahi 2007 | Unclear | Unclear | Low | Unclear | Low |
| Franz 2000 | Unclear | Unclear | Unclear | Unclear | High |
| Friel 2003 | Unclear | Unclear | Low | Low | High |
| Fujiu 2004 | Unclear | Unclear | Unclear | Unclear | Low |
| Gebresellasie 1996 | Low | Low | Low | Low | Low |
| Geltman 2001 | Unclear | Low | Low | Low | Unclear |
| Geltman 2004 | Low | Unclear | Low | Low | Unclear |
| Gokcay 2012 | Unclear | Unclear | High | Unclear | High |
| Gopaldas 1985 | Unclear | Unclear | Low | Low | Low |
| Gopaldas 1985 | Unclear | Unclear | Low | Low | Low |
| Greisen 1986 | Unclear | Unclear | Unclear | Unclear | Unclear |
| Hacıhamdioglu 2013 | Unclear | Unclear | High | Unclear | Unclear |
| Harvey 1989 | Unclear | Unclear | Low | Low | Low |
| Hathirat 1992 | Unclear | Unclear | Low | Unclear | Unclear |
| Hess 2002 | Unclear | Unclear | Low | Low | Low |
| Hettiarachchi 2008 | Unclear | Unclear | Low | Low | Low |
| Hieu 2012 | Low | Low | Low | Low | Unclear |
| Hop 2005 | Low | Low | Low | Low | Unclear |
| Htet 2019 | Low | Low | Low | Low | Low |
| Idjradinata 1993 | Low | Unclear | Low | Low | Low |
| Idjratinata 1994 | Low | Unclear | Unclear | Unclear | Unclear |
| Irigoyen 1991 | Unclear | Unclear | Low | Low | High |
| Jalambo 2018 | Unclear | Unclear | High | Low | Low |
| Kapur 2003 | Low | Unclear | Low | Unclear | Unclear |
| Kashyap 1987 | Unclear | Unclear | Low | Unclear | Unclear |
| Kashyap 1987 | Unclear | Unclear | Low | Unclear | Unclear |
| Kianfar 2000 | Unclear | Unclear | High | Unclear | Unclear |
| Kordas 2005 | Low | Low | Low | Low | High |
| Kusumastuti 2018 | Low | Low | Low | Low | Unclear |
| Lambert 2002 | Unclear | Unclear | Low | Low | Unclear |
| Latham 1990 | Unclear | Unclear | Low | Unclear | Low |
| Lawless 1994 | Low | Unclear | Low | Low | Low |
| Lee 1997 | Unclear | Unclear | Low | Unclear | Unclear |
| Leenstra 2009 | Low | Unclear | Low | Low | Low |
| Lind 2003 | Unclear | Low | Low | Low | Low |
| Lind 2004 | Low | Low | Low | Low | Low |
| López de Romaña 2005 | Low | Low | Low | Low | Low |
| Loría 1979 | Unclear | Unclear | Unclear | Unclear | Unclear |
| Lozoff 2016 | Low | Unclear | Low | Low | Low |
| Majumdar 2003 | Unclear | Unclear | Low | Unclear | High |
| Malan 2015 | Low | Unclear | Low | Low | High |
| Massaga 2003 | Low | Low | Low | Low | Low |
| Mebrahtu 2004 | Unclear | Low | Low | Low | Unclear |
| Mejía 1988 | Low | Low | High | Unclear | Unclear |
| Menendez 1997 | Low | Low | Low | Low | High |
| Menendez 2004 | Low | Low | Low | Low | High |
| Metallinos-Katsaras 2004 | Unclear | Unclear | Low | Low | Unclear |
| Mitra 1997 | Unclear | Unclear | Low | Low | Low |
| Mozaffari-Koshravi 2010 | High | Unclear | High | Unclear | Low |
| Mwanri 2000 | Low | Unclear | Low | Low | Low |
| Nagpal 2004 | Low | Unclear | Low | Low | High |
| Nair 2017 | High | High | Unclear | Unclear | Low |
| Nchito 2009 | Unclear | Unclear | Low | Low | High |

| | | | | | |
|---|---|---|---|---|---|
| Northrop-Clewes 1996 | Unclear | Unclear | Low | Low | Unclear |
| Olsen 2000 | Unclear | Unclear | Low | Low | Low |
| Olsen 2006 | Low | Low | Unclear | Unclear | Unclear |
| Paganini 2017 | Low | Low | Low | Low | Low |
| Palupi 1997 | Unclear | Unclear | Low | Low | Unclear |
| Paracha 1993 | Unclear | Unclear | Low | Unclear | Unclear |
| Perrone 1999 | Unclear | Unclear | High | Unclear | Unclear |
| Prasetyani 2017 | Low | Unclear | Low | Low | Low |
| Reeves 1985 | High | Unclear | Unclear | Unclear | High |
| Rezaeian 2014 | High | Unclear | Low | Low | Low |
| Richard 2006 | Unclear | Unclear | Low | Low | Low |
| Rosado 1997 | Unclear | Unclear | Low | Low | Low |
| Rosado 2006 | Low | Unclear | Low | Low | Unclear |
| Roschnik 2004 | Low | Unclear | High | Unclear | Unclear |
| Sarker 2008 | Low | Low | Low | Low | Unclear |
| Seshadri 1989 | Unclear | Unclear | Low | Low | Low |
| Smith 1989 | Unclear | Unclear | Low | Low | Unclear |
| Smuts 2005 | Unclear | Low | Low | Low | High |
| Smuts 2014 | Low | Unclear | Low | Low | Unclear |
| Soemantri 1989 | Unclear | Unclear | Low | Unclear | Unclear |
| Soewondo 1989 | Unclear | Unclear | Low | Low | Unclear |
| Stoltzfus 2001 | Low | Low | Low | Low | Unclear |
| Stoltzfus 2004 | Unclear | Unclear | Low | Low | High |
| Sungthong 2002 | Low | Low | Low | Low | Low |
| Sungthong 2004 | Low | Low | Low | Low | Low |
| Teshome 2017 | Low | Low | Low | Low | Unclear |
| Thibault 1993 | Unclear | Unclear | Low | Low | Unclear |
| Untoro 2005 | Unclear | Unclear | Low | Low | Low |
| van den Hombergh 1996 | Unclear | Unclear | Unclear | High | Low |
| Verhoef 2002 | Low | Low | Low | Low | Low |
| Wang 2012 | Unclear | Low | Low | Unclear | High |
| Wasantwisut 2006 | Low | Low | Low | Low | Low |
| Wieringa 2007 | Low | Low | Low | Low | High |
| Yalçin 2000 | Unclear | Unclear | High | High | High |
| Yip 1985 | High | Unclear | Unclear | Unclear | Unclear |
| Yurdakök 2004 | Unclear | Unclear | High | High | Low |
| Zavaleta 2000 | Unclear | Unclear | Low | Low | Low |
| Zhao 2004 | Unclear | Unclear | Low | Low | High |
| Ziegler 2009 | Unclear | Unclear | High | High | Unclear |
| Ziegler 2009 | Unclear | Unclear | Low | Low | Unclear |
| Zlotkin 2003 | Low | Low | High | Low | Low |
| Zlotkin 2013 | Low | Unclear | Low | Low | Low |

**Appendix 11.** Cochrane risk of bias assessment of included studies (n=129).

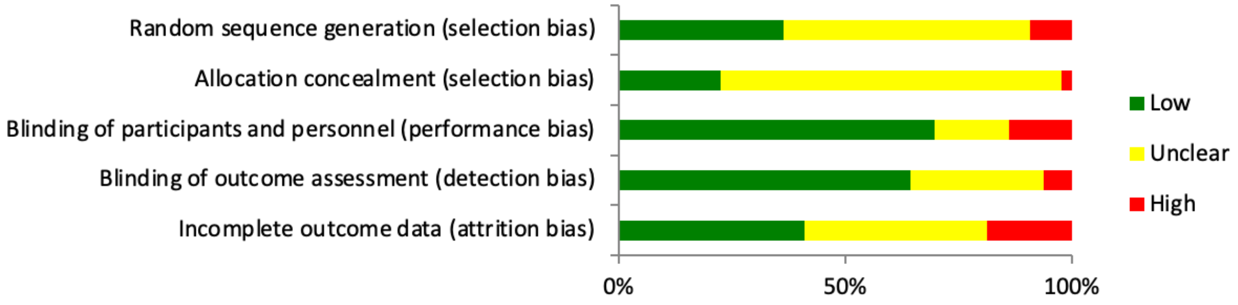

**Appendix 12.** Effect of oral iron supplementation versus placebo among children and adolescents aged <20 years among trials judged to not be at "high" risk of bias.

| | n* | Estimate type | Estimate of effect (95% CI) | p-value | $I^2$ (%) |
|---|---|---|---|---|---|
| Hemoglobin (g/L) | 105 | WMD | 6.5 (5.4, 7.6) | <0.001 | 95.5 |
| Serum ferritin (ng/mL) | 68 | WMD | 20.9 (17.4, 24.3) | <0.001 | 99.5 |
| Anemia | 42 | RR | 0.58 (0.51, 0.65) | <0.001 | 87.1 |
| Iron deficiency | 31 | RR | 0.31 (0.24, 0.41) | <0.001 | 91.1 |
| Iron deficiency anemia | 17 | RR | 0.27 (0.17, 0.44) | <0.001 | 79.9 |

* Number of trial arms randomized to iron
Abbreviations: CI, confidence interval; IRR, incidence rate ratio; PR, prevalence ratio; RR, risk ratio; SMD, standardized mean difference; WMD, weighted mean difference



Andersen CT, *et al. BMJ Global Health* 2023; 8:e010745. doi: 10.1136/bmjgh-2022-010745

**Appendix 13.** Assessment of small study bias for outcomes reported by ≥10 groups randomized to iron.

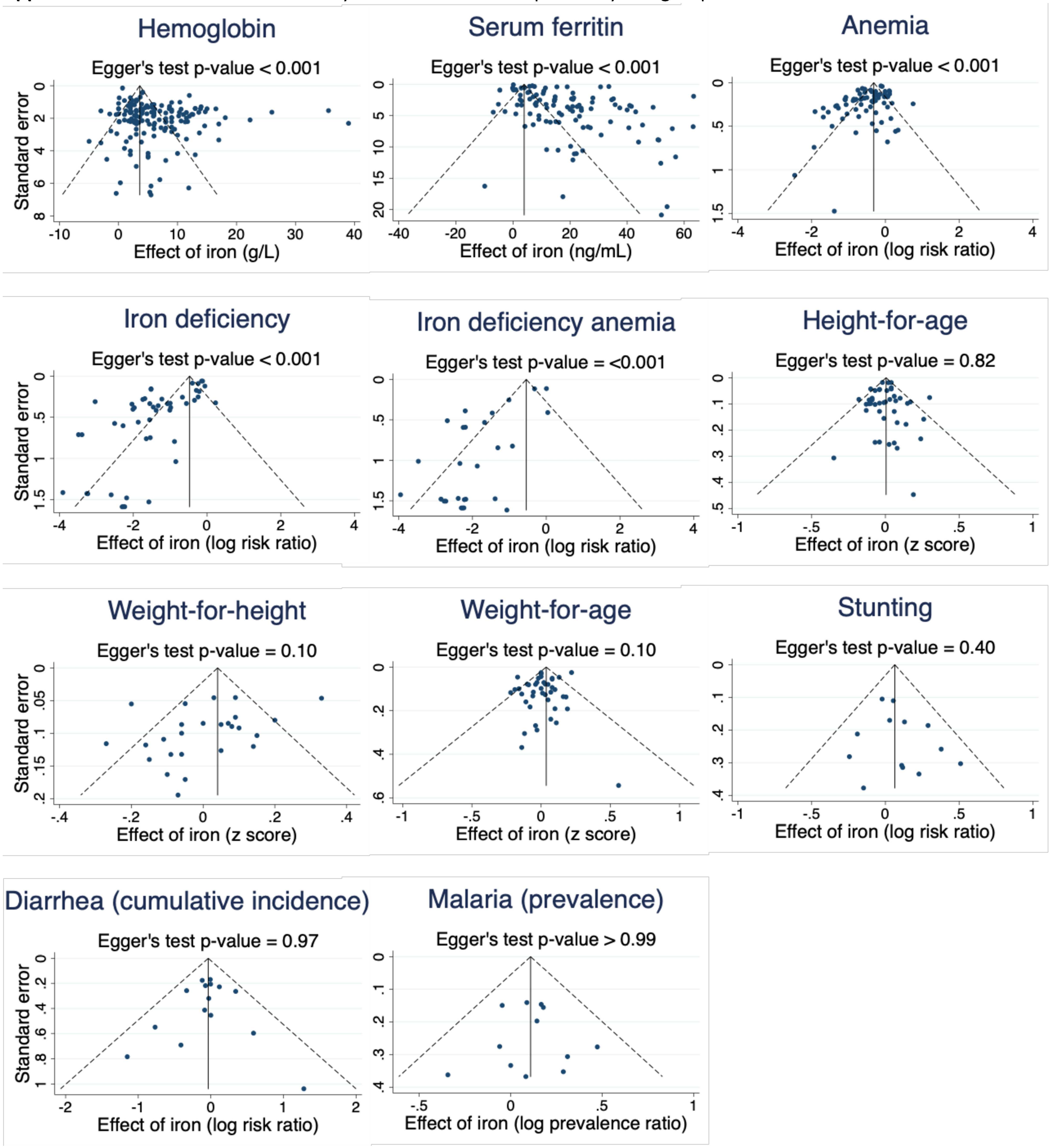

Supplement: Supplementary data [file bmjgh-2022-010745supp001.pdf]
